# Supplementary material for: R-Loops Enhance Polycomb Repression at a Subset of Developmental Regulator Genes
Source: Mol Cell. 2019 Mar 7;73(5):930–945.e4. doi: 10.1016/j.molcel.2018.12.016 (PMC6414425; doi:10.1016/j.molcel.2018.12.016)
Supplement: Document S1. Figures S1–S7 and Table S1 [file mmc1.pdf]

Molecular Cell, Volume *73*

Supplemental Information

R-Loops Enhance Polycomb Repression at a Subset of Developmental Regulator Genes

Konstantina Skourti-Stathaki, Elena Torlai Triglia, Marie Warburton, Philipp Voigt, Adrian Bird, and Ana Pombo

Skourti-Stathaki_Fig.S1

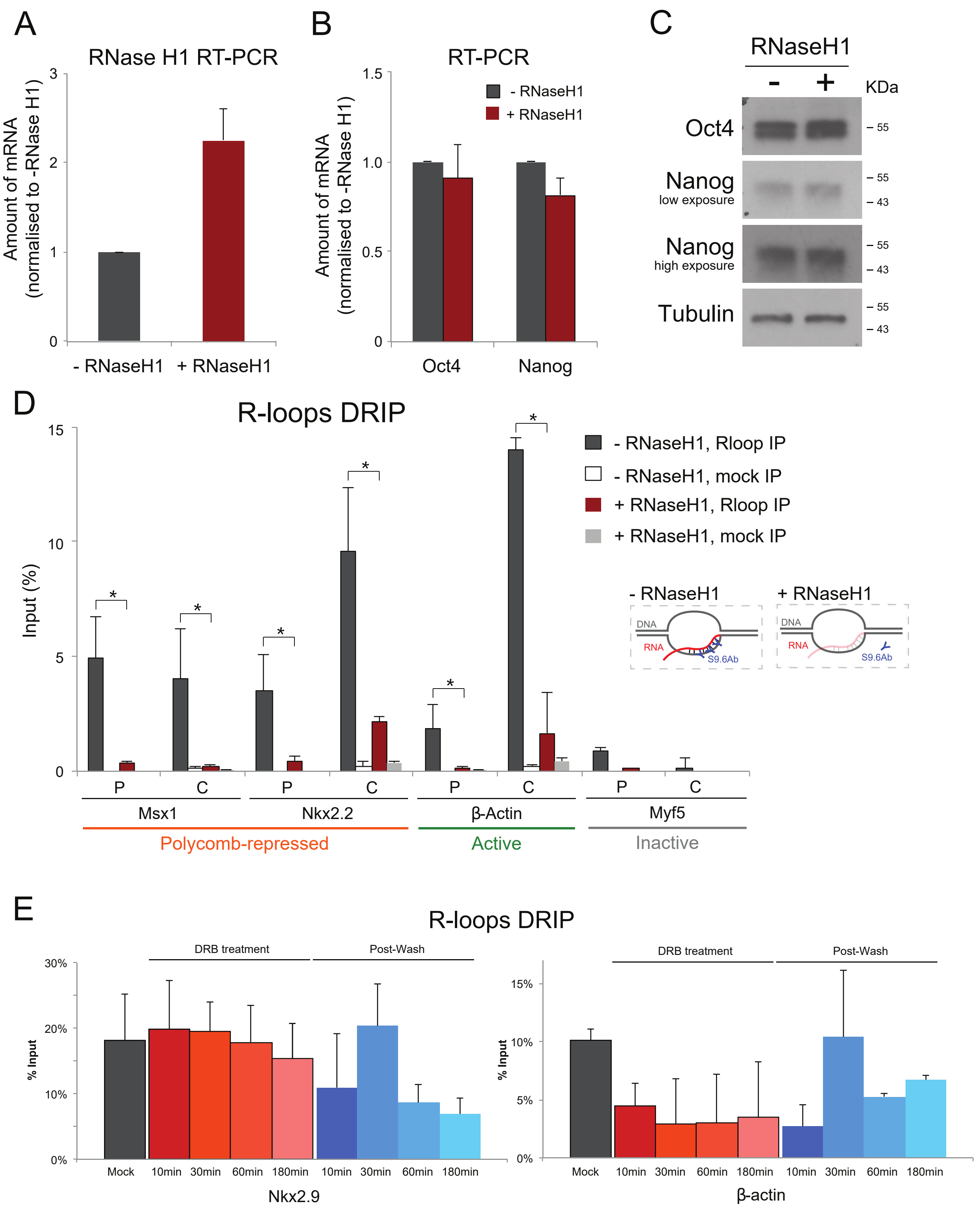

Figure S1. RNase H1 over-expression in mESCs resolves R-loops. (related to Figure 1)
(A-B) RT-qPCR analysis of RNase H1(A), Oct4 and Nanog (B) mRNA levels minus/plus RNase H1 over-expression. The amount of mRNA is normalised to control cells and was taken as 1. (C) Western blot analysis minus/plus RNase H1 over-expression. γ-tubulin was used as a loading control. (D) DRIP analysis following RNase H1 over-expression (red bars) on Polycomb-repressed, active and inactive genes. RT-qPCR and DRIP profiles are based on SD, n=3. Statistical significance was determined as in main figures. (E) DRIP analysis following DRB treatment and post-wash at the indicated time-points over the Polycomb-repressed gene *Nkx2.9* and the active *β-actin* gene. Error bars are SD, n=2.

Skourti-Stathaki_Fig.S2

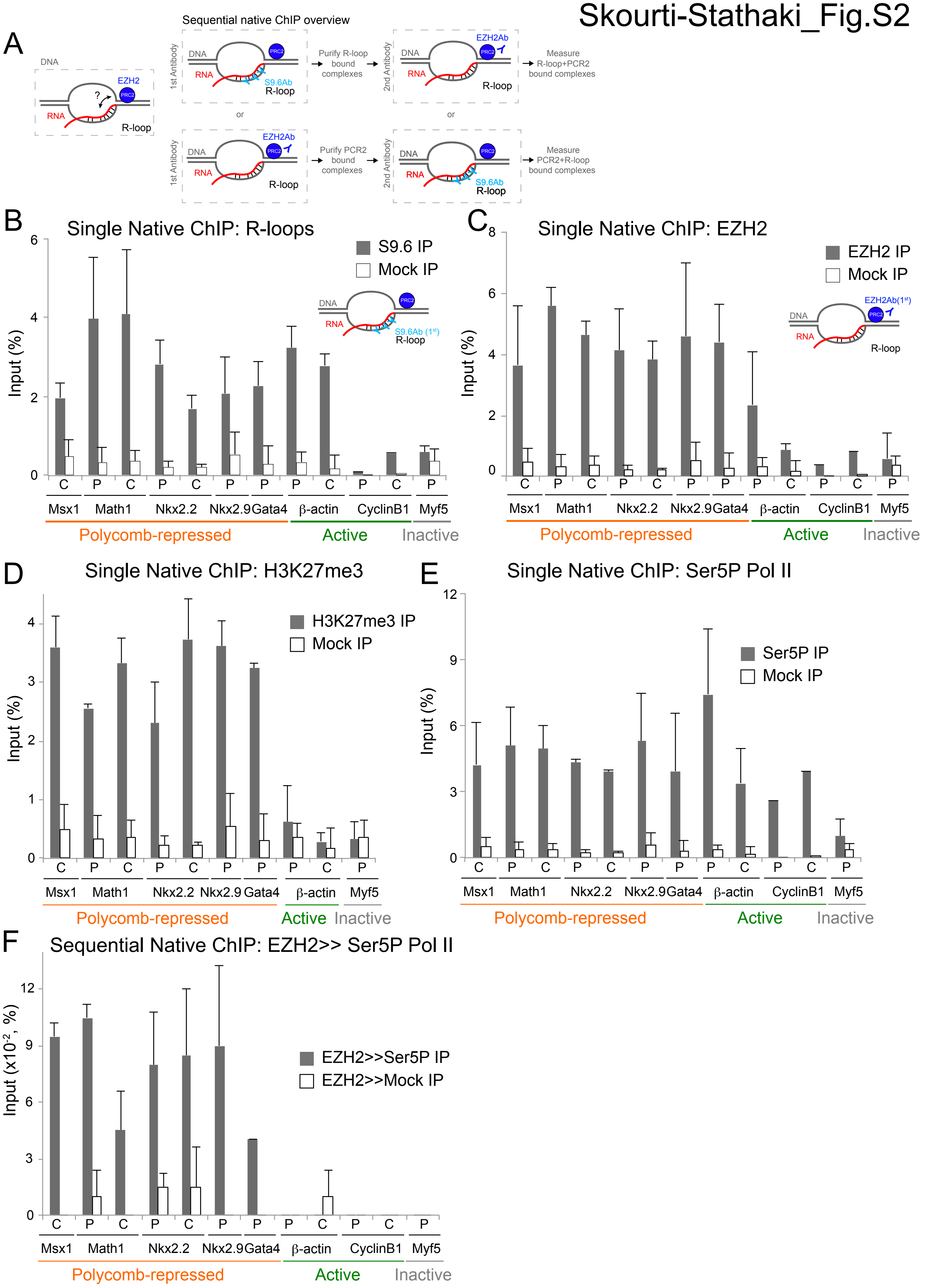

Figure S2. Validation of single native ChIP and sequential native ChIP analyses. (related to Figure 2)
(A) Schematic depicting the overview of the sequential native ChIP analysis. S9.6 (B) and EZH2 antibodies (C) native ChIP analyses on Polycomb-repressed, active and inactive genes. (D-E) Single native ChIP analyses on Polycomb-repressed genes, active and inactive genes, using H3K27me3 and Ser5P Pol II antibodies, respectively. **F.** Sequential native ChIP analysis of EZH2 with Ser5P Pol II. Error bars are SD, n=3.

Skourti-Stathaki_Fig.S3

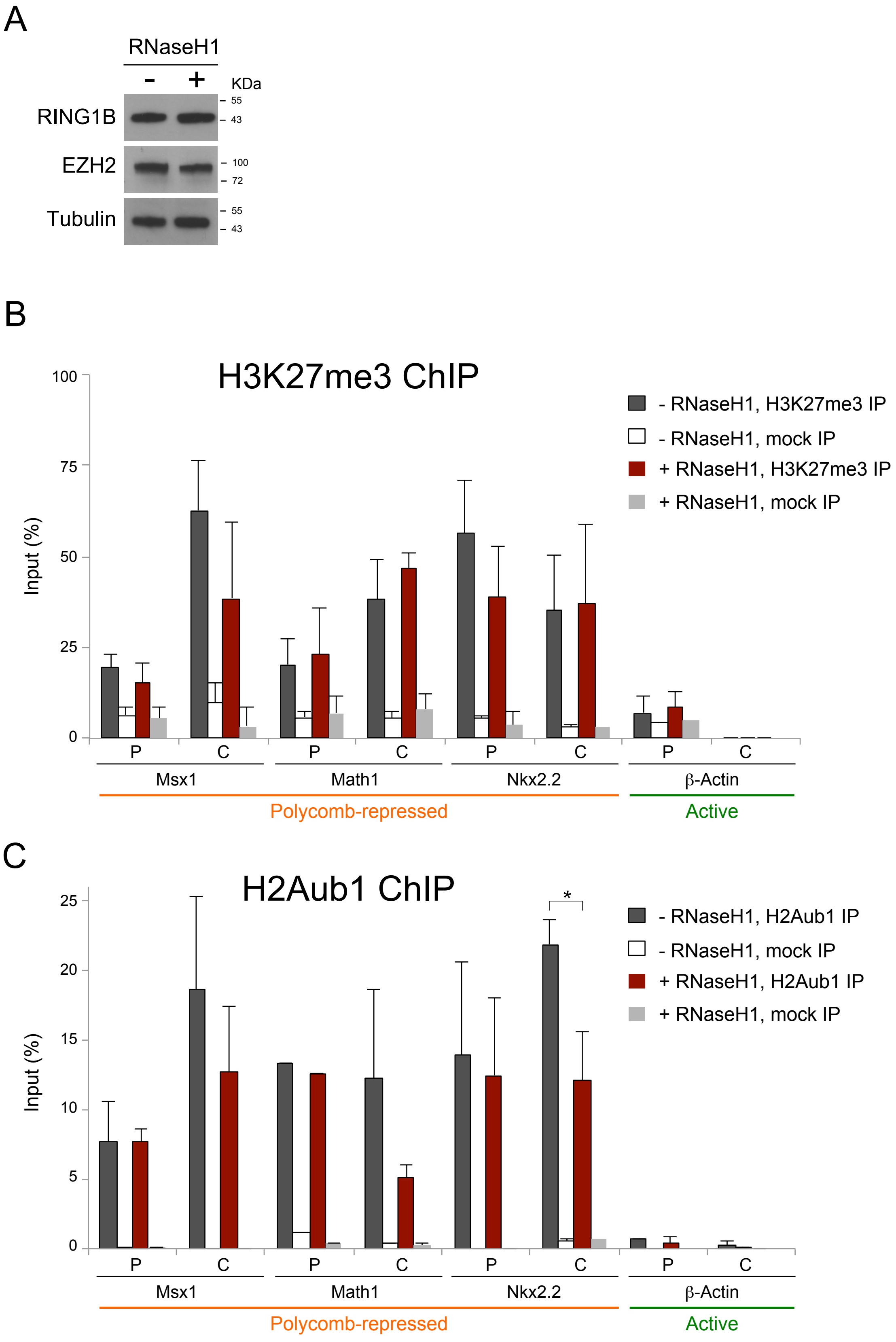

Figure S3. H3K27me3 and H2Aub1 levels upon RNase H1 over-expression. (related to Figure 2)
(A) Western blot analysis with/without RNase H1 over-expression. (B-C) ChIP analyses minus/plus RNase H1 over-expression on Polycomb-repressed and active genes, using H3K27me3 and H2Aub1 antibodies respectively. Error bars are SD, n=3 Statistical significance determined as in main figures.

Skourti-Stathaki_Fig.S4

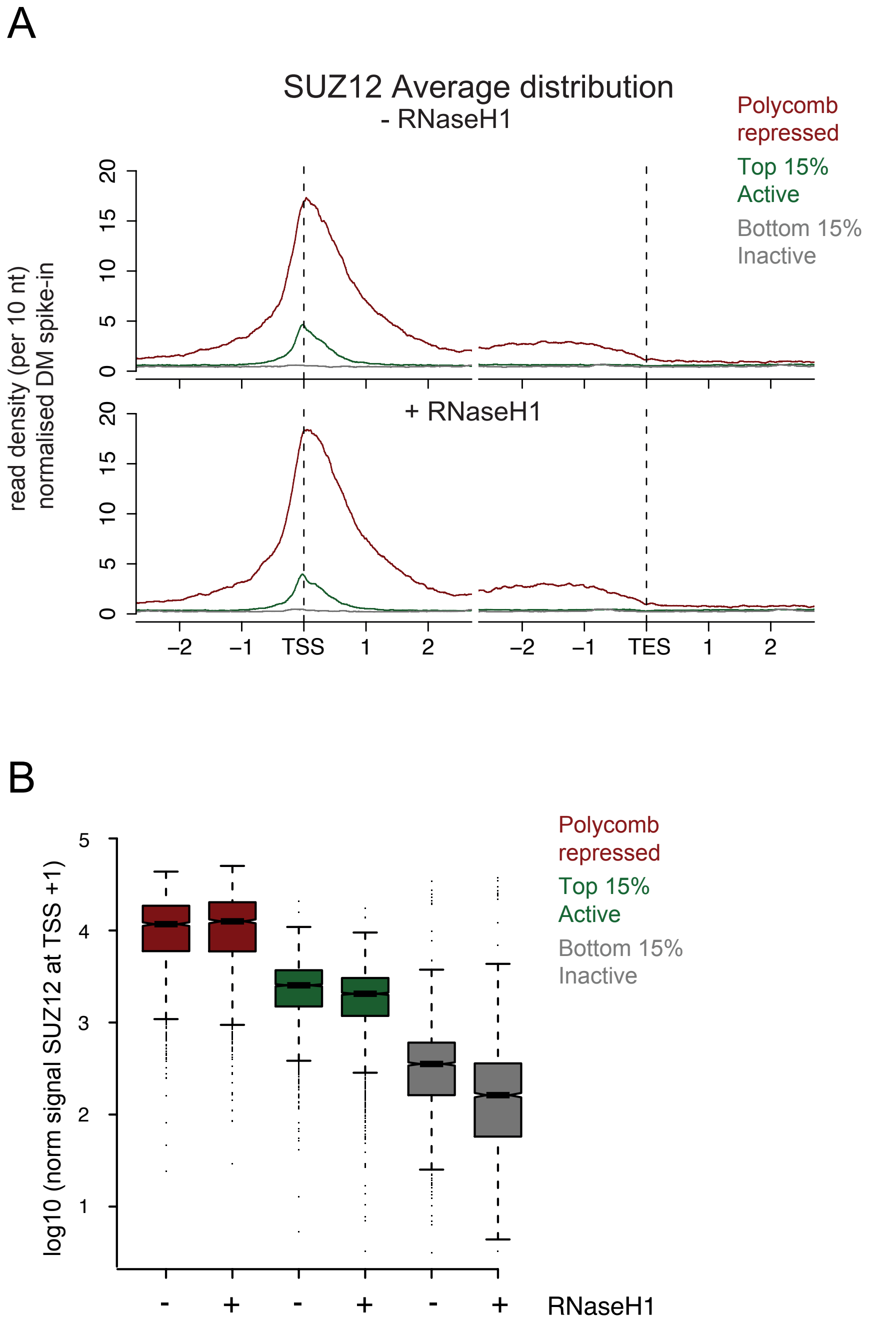

Figure S4. Genome-wide distribution of SUZ12 is only mildly affected upon R-loop resolution at Polycomb-repressed genes. (related to Figure 3)

(A) Average distribution of SUZ12 in minus/plus RNAseH1 at Polycomb-repressed genes (n=1632). Most active (Top 15%, n=2829) and least active genes (bottom 15% inactive, n=2829) are shown for comparison. (B) Boxplot with amount of signal for SUZ12 in absence (minus) or presence (plus) of RNAseH1 in 1kb centered around TSS at Polycomb repressed genes (n=1632). Most Active (Top 15%, n=2829) and least active genes (bottom 15% Inactive, n=2829) are shown for comparison. Amount of signal for both panels is normalized using Drosophila Spike-Ins (see Methods)

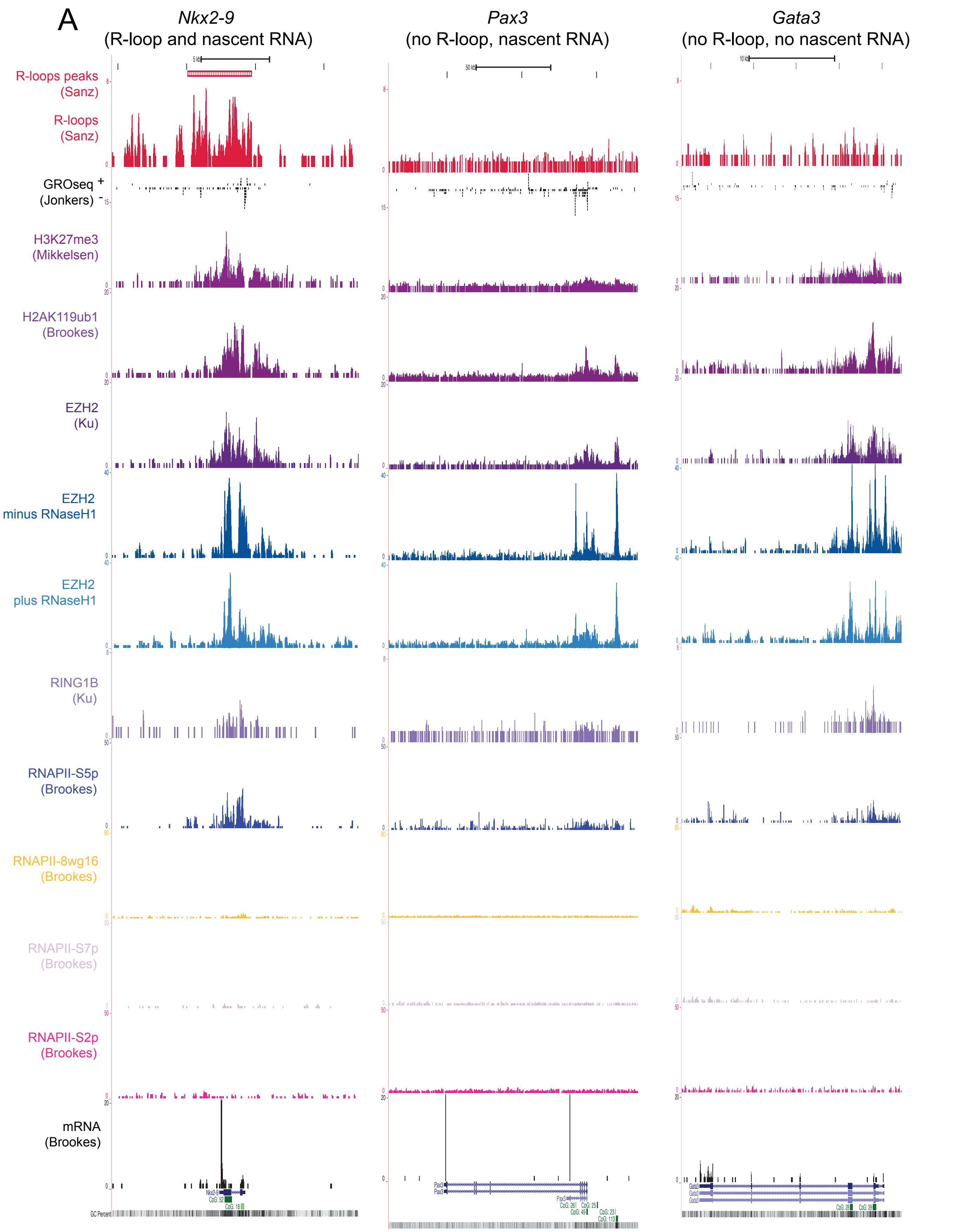

Figure S5. Representative examples of R-loop positive and R-loop negative Polycomb-repressed genes. (related to Figure 3)

UCSC browser tracks of R-loops, Polycomb, RNA Pol II and transcription at Polycomb-repressed genes with nascent RNA plus R-loops (*Nkx2.9*), nascent RNA minus R-loops (*Pax3*) and neither nascent RNA nor R-loops (*Gata3*).

Skourti-Stathaki_Fig.S6

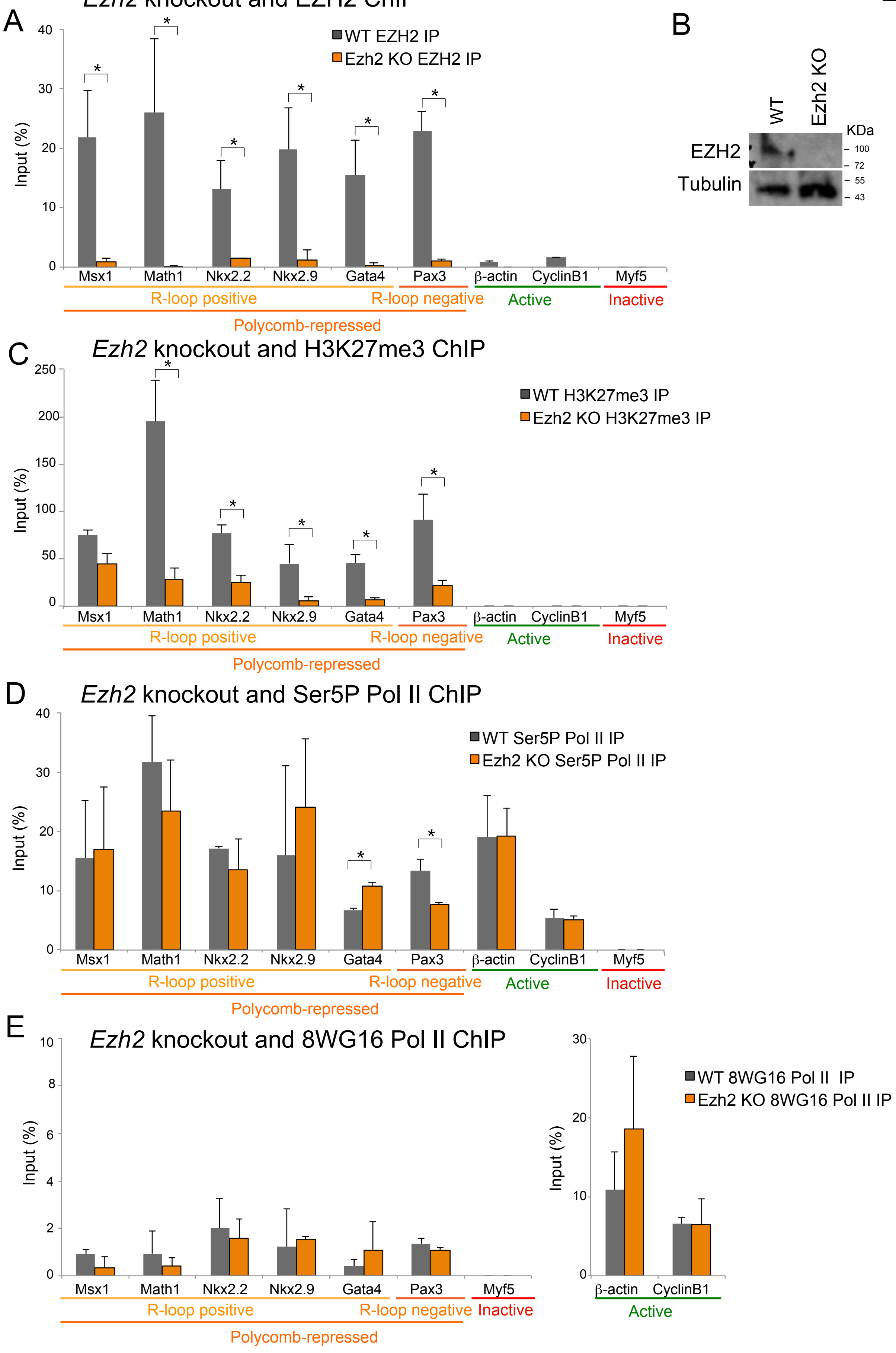

Figure S6. EZH2, H3K27me3, Ser5P and 8WG16 Pol II levels in *Ezh2* KO cells. (related to Figure 5)
(A) ChIP analysis in *Ezh2* KO cells (orange bars) using EZH2 antibody. (B) Western blot analysis in WT and *Ezh2* KO mESC probing for EZH2. γ-tubulin was used as a loading control. (C-E) ChIP analyses in *Ezh2* KO cells (orange bars) using H3K27me3 (C), Ser5P Pol II (D) and 8WG16 Pol II (E) antibodies. Error bars are SD, n=3. Statistical significance was determined as in main figures.

Skourti-Stathaki_Fig.S7

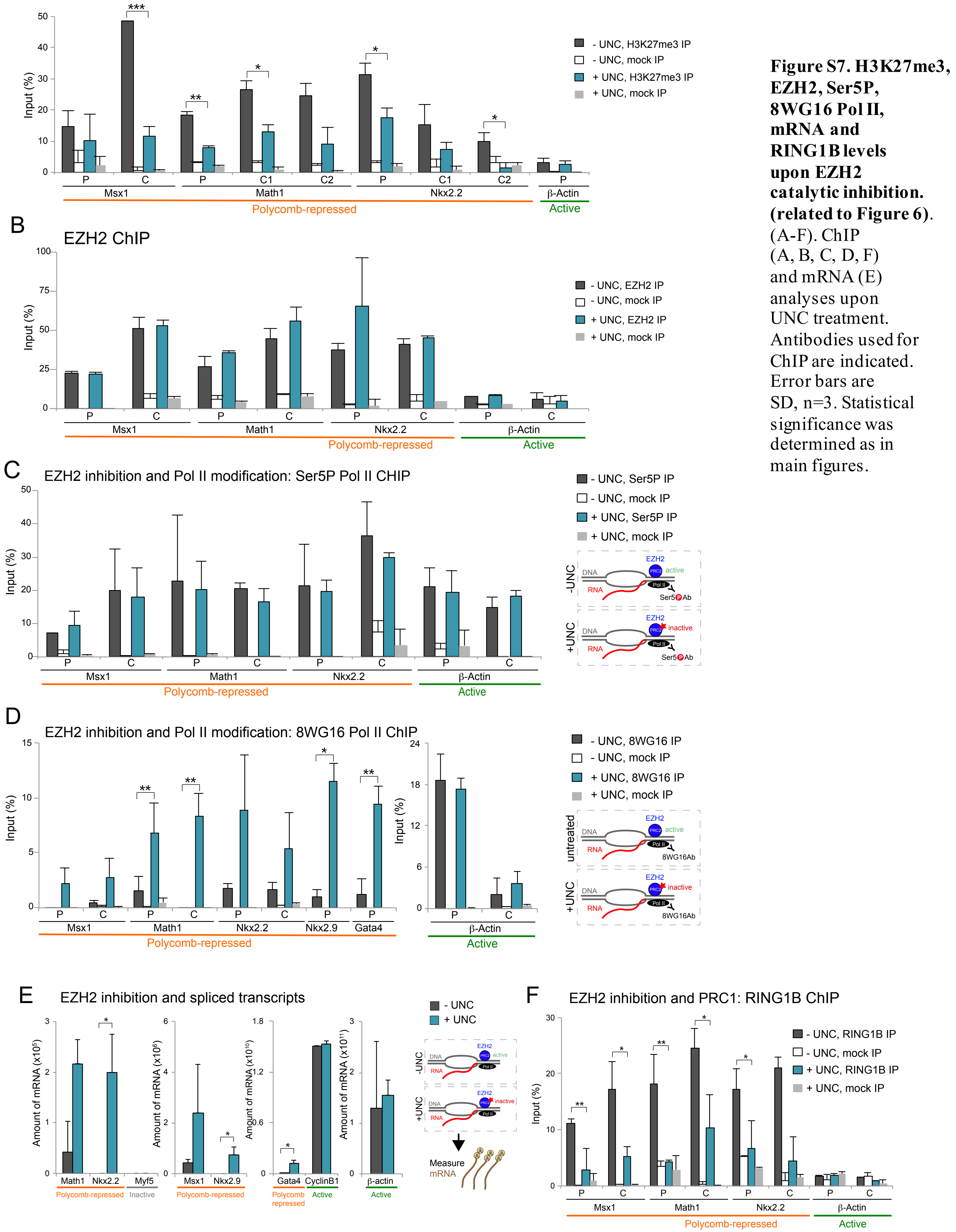

Figure S7. H3K27me3, EZH2, Ser5P, 8WG16 Pol II, mRNA and RING1B levels upon EZH2 catalytic inhibition. (related to Figure 6). (A-F). ChIP (A, B, C, D, F) and mRNA (E) analyses upon UNC treatment. Antibodies used for ChIP are indicated. Error bars are SD, n=3. Statistical significance was determined as in main figures.

Supplementary Table 1: Oligonucleotides (related to STAR Methods)

Name	Sequence (5' → 3')
**Polycomb-repressed genes**	
Msx1 P (F)	CTT AGC TAG GCG GAA AAG CTC
Msx1 P (R)	GAG AGA ACC ATT GGG CTG TG
Msx1 C1 (F)	ATC CTA GCT CTG CGG AGT TTC
Msx1 C1 (R)	TCC CCT CTT GCT AAA TCA TCC
Msx1 C2 (F)	AAA CCT GGG TGA CTT TGG ACT
Msx1 C2 (R)	AGC AGA GAC AGT GCC AAC CTA
Math1 P (F)	GGT CAG AGG AGG AAG GAA AAA
Math1 P (R)	CCC CCA ACT CTT TTA CCT CAG
Math1 C (F)	GTG AAT GGG GTA CAG AAG CAA
Math1 C (R)	TTG ATG TAG ATC TGG GCC ATC
Nkx2.2 P (F)	TAG ATA AAG GCG GGT GTT GAA
Nkx2.2 P (R)	CAG GAG ACT CAC CCC TCA AA
Nkx2.2 C1 (F)	TAC CAG CAA GGG GAG TTC TTT
Nkx2.2 C1 (R)	CCT CAT CCT CCA CAC CTA CAA
Nkx2.2 C2 (F)	TCT TCC TCA GCA TCT CCT CAA
Nkx2.2 C2 (R)	CGG TTT TGA AAT GCT GGT TTA
Nkx2.9 P (F)	TGG CAC CTT CCG GAC TTG
Nkx2.9 P (R)	AAG TGC GAG GCG CTC G
Gata4 P (F)	AAG AGC GCT TGC GTC TCT A
Gata4 P (R)	TTG CTA GCC TCA GAT CTA CGG
Msx1 nascent (F)	CGC TCG AGT TGG CCT TCT
Msx1 nascent (R)	CGG AGT CCT CCA CTT TGA CAC
Math1 nascent (F)	TGT GCG ATC TCC GAG TGA
Math1 nascent (R)	CTC GGA GGT GCC GTG TTA
Nkx2.2 nascent (F)	CGC TGC GCA GAC TCT CCT CT
Nkx2.2 nascent (R)	GAA GAG AAG CGC ATC AGG CG
Nkx2.9 nascent (F)	GTG CGC AGC CTC CTG AAT
Nkx2.9 nascent (R)	GGT CCC TCC TCC GCA CTC
Gata4 nascent (F)	GGA CTC ACG GAG ATC GCG
Gata4 nascent (R)	GGA CTC GGG GAA CCC TAC C
Msx1 spliced (F)	GCC TCT CGG CCA TTT CTC AG
Msx1 spliced (R)	CGG TTG GTC TTG TGC TTG CG
Math1 spliced (F)	GGA GAA GCT TCG TTG CAC GC
Math1 spliced (R)	GGG ACA TCG CAC TGC AAT GG
Nkx2.2 spliced (F)	TGT GCA GAG CCT GCC CCT TAA
Nkx2.2 spliced (R)	GCC CTG GGT CTC CTT GTC AT
Nkx2.9 spliced (F)	GGC CAC CTC TGG ACG CCT CG

(continues - part 2 of 3)	
Nkx2.9 spliced (R)	GCC AGC TGC GAC GAG TCT GC
Gata4 spliced (F)	GAG GCT CAG CCG CAG TTG CAG
Gata4 spliced (R)	CGG CTA AAG AAG CCT AGT CCT TGC TT
Pax3 P (F)	ACC TGT CCA CCC TTC TCT TGA
Pax3 P (R)	TCA CCC AAA GCT TGA TCA GGA
Pax3 nascent (F)	TCC CCA ACC CTT GCC TAC TAT
Pax3 nascent (R)	ATT GAG CGA TCG GAA TGA GGT
Pax3 spliced (F)	GTC CCA TGG TTG CGT CTC TAA
Pax3 spliced (R)	CTA AAC ATG CCC GGG TTC TCT
Hoxa7 P (F)	GAG AGG TGG GCA AAG AGT GG
Hoxa7 P (R)	CCG ACA ACC TCA TAC CTA TTC CTG
Hoxa7 nascent (F)	TAG ATC TTC GGG GAA CTT GGC
Hoxa7 nascent (R)	CAG AGT AGC CTT GGC CTT TCA
Hoxa7 spliced (F)	GGA AGC TGA GAG ACG TTG ACT
Hoxa7 spliced (R)	ATT TGT TGT CCG GCA GCT TTC
Mogat1 P (F)	TCC CTT TGC CTG TAG ACC TCT
Mogat1 P (R)	TCT GGC AAA AGC TCC CAA AAG
Mogat1 nascent (F)	GAC ACC ATG ACC ACA GCT CTT
Mogat1 nascent (R)	AGA TGC TCA AGT CAC ACC CAG
Mogat1 spliced (F)	GCA AGG AGG CAG AAG ATG GAA
Mogat1 spliced (R)	TCC AGG CAC GAA TAT TCC ATG A
**Active genes**	
β-actin P (F)	GAG GGG AGA GGG GGT AAA
β-actin P (R)	GAA GCT GTG CTC GCG G
β-actin C (F)	CAC CAT TCA CCA TCT TGT C
β-actin C (R)	TGA TCC ACA TCT GCT GG
CyclinB1 P (F)	GCT AGC TTG GAC AGC ACA CA
CyclinB1 P (R)	GTT CCC GTA GAA TGC GTT TC
CyclinB1 C (F)	AGT TTA GAG CCA GCC AGG ACT
CyclinB1 C (R)	GAG AAA AGC ACT GCA ATC AGG
β-actin nascent (F)	CCA CCC GCG AGC ACA
β-actin nascent (R)	CCG GCG TCC CTG CTT AC
β-actin spliced (F)	TCT TTG CAG CTC CTT CGT TG
β-actin spliced (R)	ACG ATG GAG GGG AAT ACA GC
CyclinB1 spliced (F)	TAG GGT GTC TTC TCG AAT CGG
CyclinB1 spliced (R)	ACC AAT GTC TCC AAG AGC AGT
RNase H1 (F)	GAA GGC ACA AGT GCA AGA CTC
RNase H1 (R)	TGT TCC TTC AAG GTG ATC CAG
Oct4 (F)	ACC TCA GGT TGG ACT GGG CCT A

(continues - part 3 of 3)	
Oct4 (R)	GCC TCG AAG CGA CAG ATG GT
Nanog (F)	GAA ATC CCT TCC CTC GCC ATC
Nanog (R)	CTC AGT AGC AGA CCC TTG TAA GC
**Inactive gene**	
Myf5 P (F)	GGT TGT GGT GGG ATA TGC TAA
Myf5 P (R)	GGA GTT TGG GAC TGT CTC TCT G
Myf5 C (F)	TCT GTG AGA TGG ATG GGA ACT
Myf5 C (R)	TCT TTG TGT CCC TCT CAG GTG
Myf5 nascent (F)	GGA ATA TAT AAA GAG CCC CAA CC
Myf5 nascent (R)	TTT GGG ACT GTC TCT CTG TAA TTA AC
Myf5 spliced (F)	GAT TGC TTG TCC AGC ATT GT
Myf5 spliced (R)	AGT GAT CAT CGG GAG AGA GTT
