# Supplementary material for: R-Loops Enhance Polycomb Repression at a Subset of Developmental Regulator Genes
Source: Mol Cell. 2019 Mar 7;73(5):930–945.e4. doi: 10.1016/j.molcel.2018.12.016 (PMC6414425; doi:10.1016/j.molcel.2018.12.016)
Supplement: Document S2. Article plus Supplemental Information [file mmc2.pdf]

Article

Molecular Cell

R-Loops Enhance Polycomb Repression at a Subset of Developmental Regulator Genes

Graphical Abstract

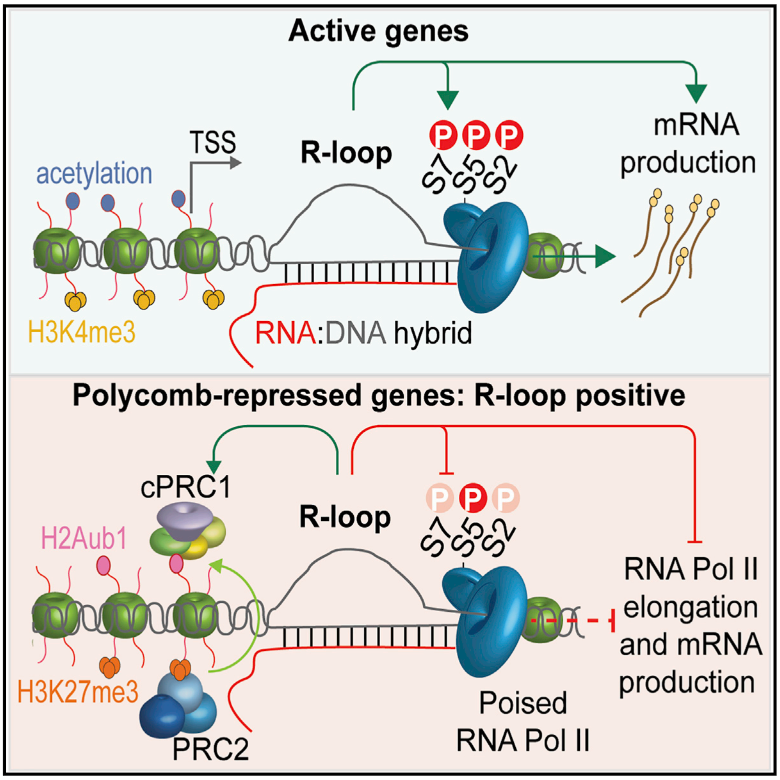

Highlights

- R-loops form at a subset of PcG target genes
- R-loops contribute to PcG recruitment genome-wide
- Loss of R-loops leads to transcriptional activation of R-loop-positive PcG targets
- R-loops and PRC1 contribute to transcriptional repression of PcG targets

Authors

Konstantina Skourti-Stathaki, Elena Torlai Triglia, Marie Warburton, Philipp Voigt, Adrian Bird, Ana Pombo

Correspondence

kskourti@staffmail.ed.ac.uk (K.S.-S.), ana.pombo@mdc-berlin.de (A.P.)

In Brief

Skourti-Stathaki and colleagues demonstrate that a subset of Polycomb-repressed genes form R-loops genome-wide. At these genes, R-loop loss leads to reduced Polycomb (PRC1 and PRC2) recruitment, enhanced polymerase II activation, and transcriptional derepression. Derepression increases in conditions of reduced PRC1 recruitment, suggesting that R-loops and PRC1 both contribute to Polycomb repression.

Skourti-Stathaki et al., 2019, Molecular Cell *73*, 930–945
March 7, 2019 © 2019 The Authors. Published by Elsevier Inc.
https://doi.org/10.1016/j.molcel.2018.12.016

CellPress

Molecular Cell
Article

R-Loops Enhance Polycomb Repression at a Subset of Developmental Regulator Genes

Konstantina Skourti-Stathaki,[1,2,6,*] Elena Torlai Triglia,[2,5] Marie Warburton,[1] Philipp Voigt,[1] Adrian Bird,[1] and Ana Pombo[2,3,4,*]

[1]Wellcome Trust Centre for Cell Biology, University of Edinburgh, EH9 3BF Edinburgh, UK
[2]Berlin Institute for Medical Systems Biology, Max Delbrueck Centre for Molecular Medicine, Berlin-Buch 13092, Germany
[3]Berlin Institute of Health, Berlin, Germany
[4]Institute for Biology, Humboldt-Universitat zu Berlin, Berlin, Germany
[5]Present address: Broad Institute of MIT and Harvard, Cambridge, MA 02142, USA
[6]Lead Contact
*Correspondence: kskourti@staffmail.ed.ac.uk (K.S.-S.), ana.pombo@mdc-berlin.de (A.P.)
https://doi.org/10.1016/j.molcel.2018.12.016

SUMMARY

R-loops are three-stranded nucleic acid structures that form during transcription, especially over unmethylated CpG-rich promoters of active genes. In mouse embryonic stem cells (mESCs), CpG-rich developmental regulator genes are repressed by the Polycomb complexes PRC1 and PRC2. Here, we show that R-loops form at a subset of Polycomb target genes, and we investigate their contribution to Polycomb repression. At R-loop-positive genes, R-loop removal leads to decreased PRC1 and PRC2 recruitment and Pol II activation into a productive elongation state, accompanied by gene derepression at nascent and processed transcript levels. Stable removal of PRC2 derepresses R-loop-negative genes, as expected, but does not affect R-loops, PRC1 recruitment, or transcriptional repression of R-loop-positive genes. Our results highlight that Polycomb repression does not occur via one mechanism but consists of different layers of repression, some of which are gene specific. We uncover that one such mechanism is mediated by an interplay between R-loops and RING1B recruitment.

INTRODUCTION

During transcription, nascent RNA can hybridize with the DNA template strand, leaving the non-template DNA strand single stranded. These structures are called R-loops, and their persistent formation can cause deleterious effects on genome integrity, possibly due to the unpaired single-stranded DNA (ssDNA) (Aguilera and García-Muse, 2012; Hamperl and Cimprich, 2014; Skourti-Stathaki and Proudfoot, 2014).

Even though R-loops have the potential to form over a large proportion of the genome, they are not a simple consequence of transcription. They occur at specific, conserved loci as a result of a complex interplay of the DNA sequence, transcription, topology, and chromatin environment (Chédin, 2016). At active mammalian protein-coding genes with unmethylated CpG-island promoters, R-loops are enriched over promoters and termination sites and enhance activation, and they are linked with histone marks of active transcription, such as mono- and tri-methylation of lysine 4 of histone H3 (H3K4me1/3) and H3 acetylation (Ginno et al., 2012, 2013; Sanz et al., 2016). R-loops can act as transcriptional activators, but they can also induce transcriptional repression in different cell types and via various mechanisms (Nakama et al., 2012; Castellano-Pozo et al., 2013; Powell et al., 2013; Sun et al., 2013; Skourti-Stathaki et al., 2014). This "dual" function of R-loops in activation or repression strongly suggests that R-loop formation can have different roles and mechanisms in different contexts.

The Polycomb group (PcG) proteins are major epigenetic regulators of transcriptional repression, and they are required to silence CpG-rich developmental regulator genes in embryonic stem cells (ESCs) and maintain patterns of gene expression established during cell commitment (Margueron and Reinberg, 2011; Di Croce and Helin, 2013). They assemble in two major multi-subunit complexes: Polycomb repressive complex 1 (PRC1) and PRC2. The catalytic components of PRC1 and PRC2 are RING1B and EZH2, respectively. RING1B monoubiquitinylates histone H2A in lysine 119 (H2Aub1), and EZH2 is a methyltransferase responsible for the di- and tri-methylation of H3 in lysine 27 (H3K27me2/3).

The mechanisms of Polycomb-mediated transcriptional repression are not fully understood. Despite their roles in repression, PcG-target genes in mouse embryonic stem cells (mESCs) display the active chromatin mark H3K4me3 (Azuara et al., 2006; Bernstein et al., 2006a; Voigt et al., 2012), RNA polymerase II (Pol II), and general transcription factors (Breiling et al., 2001; Dellino et al., 2004; Chopra et al., 2009). Pol II is detected over promoters and coding regions of PcG-repressed genes and exhibits serine5 phosphorylation (Ser5P) of its C-terminal domain (CTD), but not Ser2P or Ser7P, the latter being markers of productive transcriptional elongation (Stock et al., 2007; Brookes et al., 2012; Tee et al., 2014). Consistent with the presence of poised Pol II, low levels of nascent transcripts, but no significant amounts of mRNA, have been detected at some PcG targets

 © 2019 The Authors. Published by Elsevier Inc.
This is an open access article under the CC BY license (http://creativecommons.org/licenses/by/4.0/).

(Guenther et al., 2007; Stock et al., 2007; Kanhere et al., 2010; Mikkelsen et al., 2007).

PcG recruitment to its target genes remains a complex pathway, as different mechanisms and factors have been invoked (reviewed in Blackledge et al., 2015). For example, transcription itself plays a role, as gene silencing alone can promote PRC2 recruitment to CpG island promoters (Ku et al., 2008; Riising et al., 2014). Recruitment of the canonical PRC1 to its targets is proposed to occur through a hierarchical process by prior deposition of H3K27me3 by PRC2 (Wang et al., 2004; Boyer et al., 2006). However, non-canonical PRC1 (which contains RYBP instead of CBX) can also be targeted to CpG islands by KDM2B lysine demethylase (Farcas et al., 2012; He et al., 2013) and recruit PRC2 via H2Aub1 (Blackledge et al., 2014; Cooper et al., 2014).

The presence of R-loops at PcG target genes has so far been explored in human ESCs, where a positive correlation was detected based on bioinformatic analysis (Ginno et al., 2013), and in differentiated mouse fibroblasts (NIH 3T3 line), where a negative correlation was reported (Sanz et al., 2016). It therefore remains unclear whether and how PcG-repression mechanisms are regulated by R-loops.

Here, we have used mESCs to explore the contribution of R-loops to PcG-repression mechanisms. We show that R-loops form at PcG targets and R-loop loss leads to deficient PcG recruitment and an altered poised state of Pol II, resulting in gene derepression. Genome-wide analyses show that R-loop formation is not a trivial consequence of low transcription levels and occurs only at a subset of PcG-repressed genes. Constitutive EZH2 (PRC2) knockout alone is not sufficient to affect R-loops or RING1B (PRC1) recruitment and does not lead to transcriptional derepression of R-loop-positive genes. In contrast R-loop removal in these conditions causes gene activation and reduced RING1B recruitment. Upon inhibition of EZH2 catalytic activity, R-loops and RING1B can repress PcG targets. Our results uncover an unanticipated interplay between R-loops and PRC1 recruitment that contributes to PcG repression.

RESULTS

R-Loops Form over PcG-Repressed Genes

To investigate whether R-loops play a role in the PcG-mediated transcriptional silencing, we measured their presence over a panel of PcG target genes using DNA-RNA immunoprecipitation (DIP or DRIP) analysis (Skourti-Stathaki et al., 2011; Ginno et al., 2012, 2013; Skourti-Stathaki et al., 2014; Sanz et al., 2016) in mESCs. We chose five previously characterized genes, namely *Msx1*, *Math1*, *Nkx2.2*, *Nkx2.9*, and *Gata4*. These genes have well-annotated CpG island promoters, are GC-rich throughout their promoters and coding regions, and in mESCs are co-occupied by PRC1, PRC2, and poised Ser5P Pol II (Stock et al., 2007; Brookes et al., 2012; Ferrai et al., 2017). Native nuclear extracts were immunoprecipitated with the RNA-DNA-hybrid-specific antibody, S9.6 (Boguslawski et al., 1986), and the purified DNA was analyzed using primers positioned over the promoter (P) regions containing transcription start sites (TSSs) and within coding (C) regions at gene bodies. As a positive control, we used the highly expressed active gene *β-actin*, which forms R-loops over P and C regions (Skourti-Stathaki et al., 2011, 2014). As negative controls, we used the active gene *CyclinB1* that does not form R-loops (Skourti-Stathaki et al., 2014) and the gene *Myf5* that is neither expressed nor associated with PcG or Pol II in mESCs (Stock et al., 2007; Brookes et al., 2012). As expected, R-loops were enriched at *β-actin* but were not detected over *CyclinB1* or *Myf5*. Remarkably, R-loops were specifically enriched at both P and C regions at all five PcG-repressed genes (Figure 1A).

To further assess R-loop presence and confirm their specificity, we overexpressed RNase H1 *in vivo*, an enzyme that specifically degrades RNA in RNA-DNA hybrids without cleaving the single-stranded RNA (ssRNA). We transfected mESCs for 48 h with a construct expressing GFP-tagged RNase H1 (Cerritelli et al., 2003) (Figure S1A). Importantly, mESCs retained expression of the pluripotency markers *Oct4* and *Nanog* upon transfection (Figures S1B and S1C). DRIP analysis following RNase H1 overexpression showed loss of R-loop signals over PcG-repressed and active genes (Figure S1D), confirming that they are bona fide RNA-DNA hybrids.

We then sought to investigate the effects of transcription on R-loop formation and turnover at PcG-repressed genes as compared to active genes. We treated cells with 5,6-dichloro-1-β-D-ribofuranosyl-benzimidazole (DRB), a CDK9 inhibitor that interferes with transcriptional elongation by Pol II. R-loops were then measured over a PcG-repressed (*Nkx2.9*) and an active gene (*β-actin*) at specific time points after DRB treatment and post-wash conditions. As shown in Figure S1E, R-loops rapidly decreased over *β-actin* after 10 min of DRB treatment and they reappeared 30 min post-wash. This argues for a dynamic resolution and formation of R-loops over active genes, as previously reported (Sanz et al., 2016). Strikingly, R-loops over *Nkx2.9* failed to resolve even after 3 h of DRB treatment, suggesting that R-loops over PcG-repressed genes are more stable than those formed over active genes and might therefore indicate a different function of R-loops in PcG targets.

Loss of R-Loops Leads to Derepression of PcG Target Genes

Next, we investigated whether R-loop formation contributes to PcG-repression mechanisms by studying the transcriptional profiles of PcG-repressed and active genes upon R-loop removal. First, we assessed unprocessed (non-spliced and non-polyadenylated) transcripts by synthesizing cDNA from total RNA using reverse primers positioned over the first intron. Low levels of nascent transcripts could be detected over PcG-repressed genes (Figure 1B), as previously shown (Guenther et al., 2007; Stock et al., 2007; Kanhere et al., 2010). Notably, selective R-loop removal by RNase H1 overexpression led to an increase of nascent transcripts specifically over PcG-repressed genes, showing that R-loops contribute to their transcriptional silencing. In contrast, loss of R-loops over the active gene *β-actin* caused a mild decrease in the amount of nascent RNA (Figure 1B, right panel), consistent with the known transcriptional activator role of R-loops at some active genes (Skourti-Stathaki et al., 2011; Ginno et al., 2012, 2013; Sanz et al., 2016). The opposite effect of R-loop loss over PcG-repressed and active

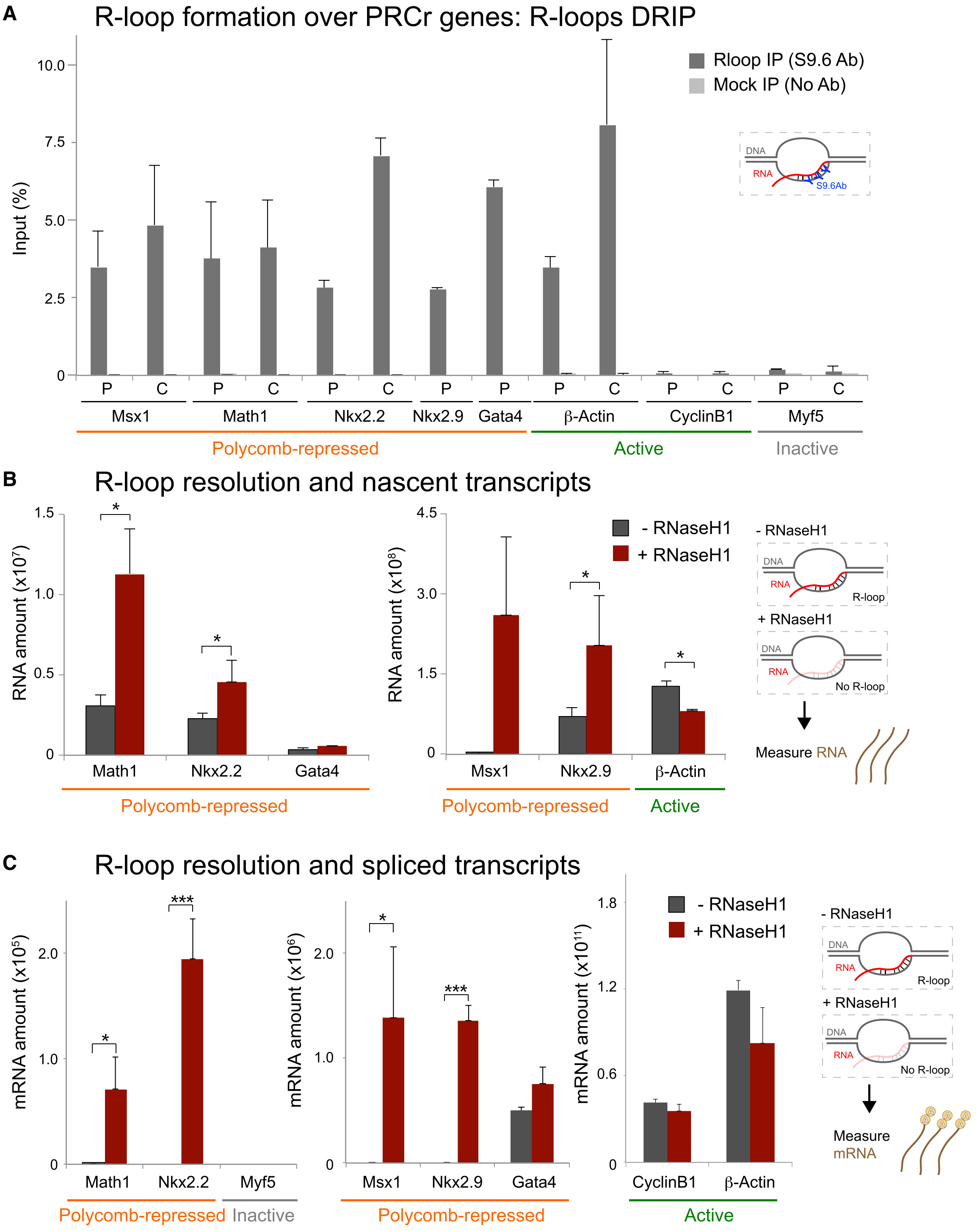

Figure 1. R-Loops Form over PcG-Repressed Genes, and Their Selective Removal Causes Transcriptional Activation

(A) DRIP analysis using the RNA-DNA hybrid antibody S9.6 over Polycomb group (PcG)-repressed, active, and inactive genes. Primer regions to promoter (P) and coding (C) regions are indicated.

(B) qRT-PCR analysis of total RNA performed on PcG-repressed and active genes with or without overexpression of RNase H1.

(C) Detection of spliced transcripts upon RNase H1 overexpression on PcG-repressed, inactive, and active genes.

Error bars represent SD; n = 3–4. *p < 0.05, **p < 0.01, ***p < 0.001.

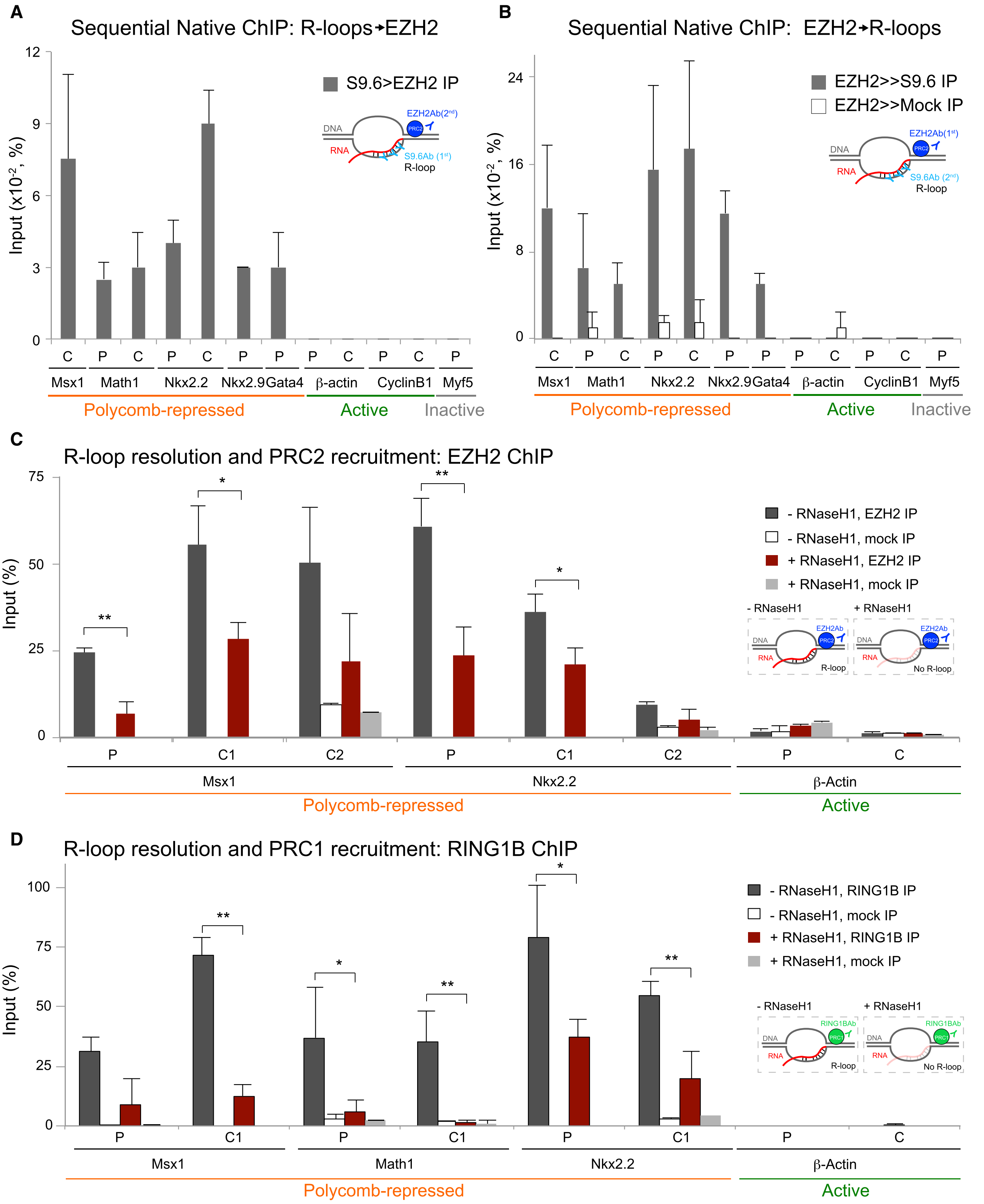

(legend on next page)

genes argues for a specific effect of RNase H1 overexpression on targeting the R-loop structure alone, rather than nascent RNAs, and a repressive role for R-loops at PcG targets. Moreover, it indicates distinct mechanisms of R-loop function at different gene groups.

We then tested whether R-loop removal is sufficient for transcript maturation and increased mRNA expression. To probe for poly-adenylated transcripts, we reverse-transcribed RNA using oligo-dT primers and amplified the cDNA using primers spanning the spliced junction between exon 1 and exon 2 for each gene (Figure 1C). We confirmed that no mRNA was detected over PcG-repressed genes in mESCs prior to R-loop removal, as expected. R-loop depletion led to the detection of spliced poly-adenylated transcripts from PcG-repressed genes, suggesting that R-loops are required for full PcG repression. Depletion of R-loops led to a mild depletion of spliced transcripts at the *β-actin* gene, whereas *CyclinB1* mRNA levels were unchanged.

R-Loops Co-occupy Chromatin with PcG Enzymes and Contribute to Their Recruitment

To further explore the mechanisms by which R-loop formation at PcG targets promote transcriptional repression, we asked whether R-loops and PcG enzymes simultaneously co-occupy chromatin using sequential native chromatin immunoprecipitation (ChIP) (Figure S2A). First, we performed single native ChIP analyses and confirmed the associations on chromatin of R-loops, EZH2, H3K27me3, and Ser5P Pol II are captured in native conditions (Figures S2B–S2E).

Sequential native ChIP revealed that EZH2 co-occupies with R-loop chromatin, independent of the immunoprecipitation order (Figures 2A and 2B). No DNA was recovered over *β-actin*, as expected, confirming that there was no detectable carryover from the first ChIP with the S9.6 antibody. Negative control genes (*CyclinB1* and *Myf5*) showed no enrichment. We also confirmed co-association of EZH2 with Ser5P Pol II in native conditions (Figure S2F), which was shown previously with cross-linked chromatin (Brookes et al., 2012). These results suggest that R-loops and PcG coincide on chromatin via indirect or direct interactions.

Next, we tested whether recruitment of PcG enzymes was altered after RNase H1 overexpression. We confirmed the occupancy of EZH2 and RING1B at both P and C regions of our panel of PcG-repressed genes, but not at active genes in control mESCs. Upon R-loop resolution, EZH2 and especially RING1B occupancy was reduced at PcG-target genes (Figures 2C and 2D), although the total levels of these proteins were unaffected (Figure S3A), suggesting that R-loops facilitate or stabilize the binding of PcG enzymes on their targets.

To test whether H3K27me3 and H2Aub1, the chromatin modifications instigated by PcG, are also affected upon R-loop removal, we performed ChIP analyses following RNase H1 overexpression (Figures S3B and S3C). RNase H1 overexpression had a minimal effect on these chromatin marks, possibly due to the stability and low turnover levels of chromatin marks during the short window of RNaseH1 overexpression (Kouskouti and Talianidis, 2005; Ferrari and Strubin, 2015). Interestingly, these conditions resulted in gene derepression upon loss of PRC occupancy without depletion of H3K27me3 and H2Aub1. Derepression may result from PRC functions that do not involve histone modifications and may therefore relate to other processes, such as chromatin condensation (Eskeland et al., 2010).

R-Loops Contribute to PcG Recruitment Genome-wide

To explore more globally which PcG-repressed genes are regulated by R-loop formation, we tested which genes lose EZH2 after R-loop depletion by performing EZH2 ChIP sequencing (ChIP-seq) on mock-transfected cells and cells overexpressing RNase H1 (Figures 3A and 3B). Consistent with the single-gene ChIP results, the average distribution of EZH2 occupancy at PcG-repressed genes decreased upon R-loop resolution, confirming the dependency of PcG occupancy levels on R-loops (Figure 3A). This effect was not observed for silent genes that are not silenced through PcG repression (inactive genes) and was less pronounced for highly expressed active genes (Figure 3B). In contrast the recruitment of SUZ12, a non-catalytic subunit of PRC2, was almost unchanged over PcG-repressed genes upon R-loop resolution in our experimental setting (Figure S4).

R-Loops Form at a Subset of PcG-Repressed Genes but Are Not an Inherent Feature of Low-Level Transcription

To explore the extent to which R-loops form at PcG-repressed genes genome-wide, we re-analyzed published genome-wide DRIP-seq dataset of R-loops in mESCs (Sanz et al., 2016). R-loops were detected at 76% of active genes in mESCs and at a minor proportion of inactive genes (Figure 3C). R-loops were also detected at 409 PcG targets, raising the possibility that R-loop formation contributes to PcG repression only at a subset of genes. Next, we sought to investigate whether R-loop detection could simply result from low-level transcription detected at PcG-repressed genes. We mined a published global run-on (GRO-seq) dataset from mESCs (Jonkers et al., 2014) and found that the R-loop-positive PcG targets are transcribed at levels similar to those of R-loop-negative PcG targets (Figure 3D). We then asked what proportion of PcG-repressed genes generating nascent RNA forms R-loops and found that 42% of them generate nascent RNA detectable with GRO-seq, and 29% of these genes form R-loops (196 genes, reads per kilobase of transcript per million mapped reads [RPKM] > 0.1; Figure 3E). These results suggest that R-loops are not an inherent feature of low-level transcription.

We then investigated whether R-loops contribute to PcG repression at genes where they specifically form. We assessed transcriptional repression and PcG recruitment in single genes that do not show detectable R-loops in the published DRIP-seq

Figure 2. R-Loops Co-occupy with PcG Proteins on Chromatin and Contribute to Their Recruitment

(A and B) Sequential native ChIP shows co-association of R-loops with EZH2 at PcG-repressed genes (A) and vice versa (B).

(C and D) EZH2 (C) and RING1B (D) ChIP analyses on PcG-repressed and active *β-actin* genes upon RNase H1 overexpression. Regions C1/C2 correspond to C regions 300–400 and 700–800 bp downstream of the TSSs, respectively.

Error bars represent SD; n = 3. *p < 0.05, **p < 0.01, ***p < 0.001.

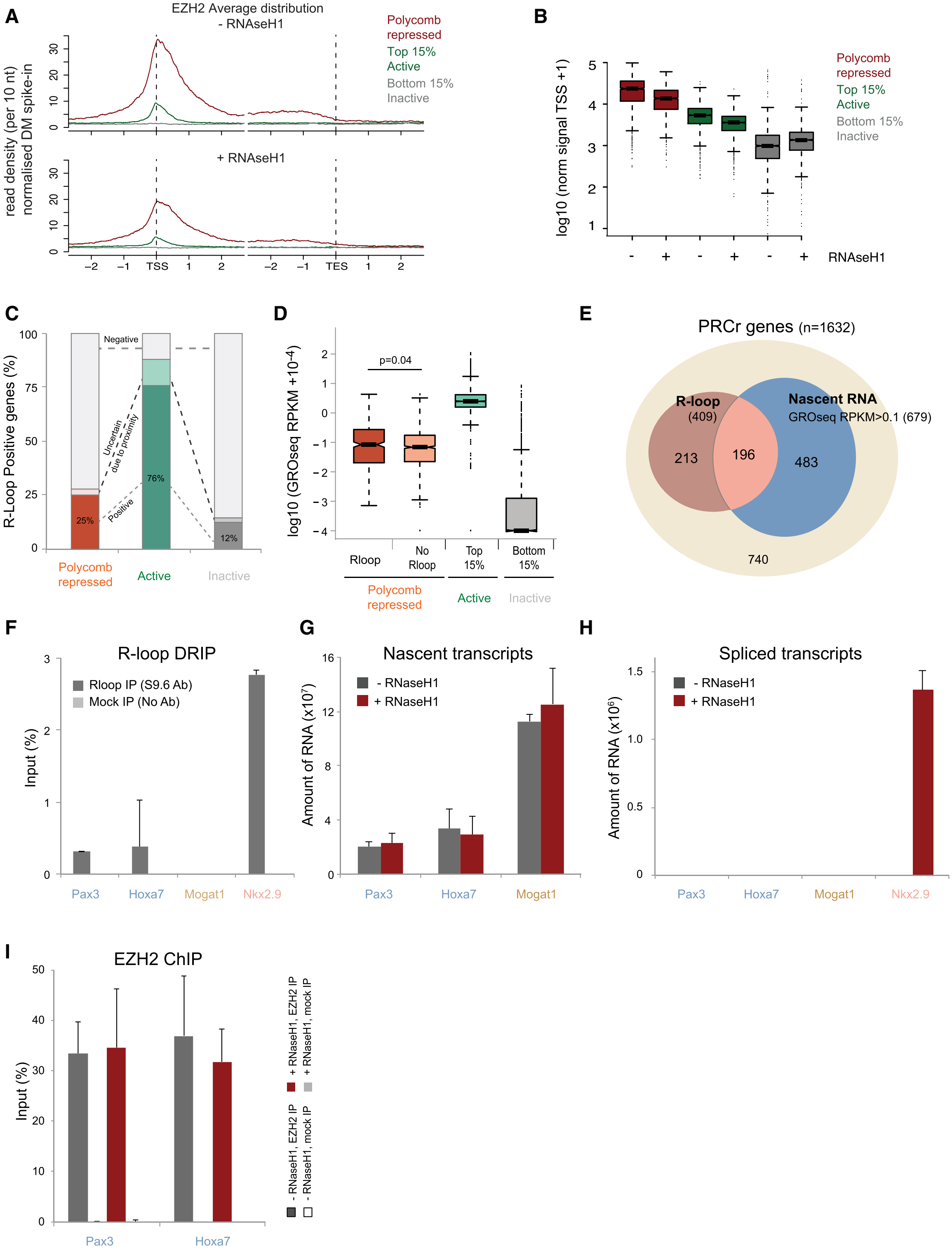

Figure 3. R-Loops Contribute to PcG Recruitment Genome-wide and Form at a Subset of PcG-Repressed Genes

(A) Average distribution of EZH2 at PcG-repressed genes (n = 1,632) minus or plus RNaseH1 overexpression. The most (top 15%, n = 2,829) and least active genes (bottom 15% inactive, n = 2,829) are shown for comparison.

(B) Boxplot with amount of signal for EZH2 minus or plus RNaseH1 in 1-kb windows centered on TSSs of Polycomb-repressed genes.

(legend continued on next page)

data but exhibit nascent RNA detected either by GRO-seq or by alternative published RNA techniques. *Hoxa7* is a gene that shows nascent RNA in the GRO-seq, and *Pax3* is a gene with nascent RNA as detected previously by northern blot (Kanhere et al., 2010). We chose *Mogat1* as a gene with no nascent RNA signal, based on the GRO-seq analysis and no detectable R-loop signal according to DRIP-seq. We employed DRIP experiments and found very low or absent R-loop signals over these genes compared to the R-loop-positive *Nkx2.9* (Figure 3F). We then confirmed the presence of nascent RNA for *Hoxa7* and *Pax3* genes, but unexpectedly, we also detected transcripts in *Mogat1*, which had no detectable GRO-seq signal (Figure 3G, gray bars). This implies that GRO-seq analysis, which is designed to detect products of actively elongating polymerases, can fail to detect low levels of nascent RNA in some PcG-repressed genes. Importantly, R-loop removal by RNase H1 overexpression did not affect either nascent or processed transcripts or the EZH2 recruitment over these R-loop-negative genes (Figures 3G–3I), unlike the deregulation observed at R-loop-positive genes (Figures 1 and 2). These results collectively highlight that R-loops contribute to PcG repression mechanisms only at genes where they specifically form. Furthermore, we found that the presence of low levels transcription is not sufficient to cause a gene to be R-loop positive. Representative examples of chromatin occupancy and transcription UCSC profiles at PcG-repressed genes with and without nascent RNA and R-loops are shown in Figure S5.

Selective Removal of R-Loops Leads to an Activated Pol II State over PcG-Repressed Genes

To further dissect the mechanism of transcriptional activation mediated by loss of R-loops at PcG target genes (Figures 1B and 1C), we examined the effect of R-loop loss on Pol II activation. Pol II at PcG-repressed genes exists in a poised state, characterized by the exclusive presence of Ser5P (Stock et al., 2007; Brookes et al., 2012; Tee et al., 2014). In contrast, at active genes, Ser5P and Ser7P mark active gene promoters and are associated with transcriptional initiation and early elongation, whereas Ser2P is associated with productive elongation and termination (Hsin and Manley, 2012; Harlen and Churchman, 2017).

To determine whether the transcriptional activation of PcG target genes observed upon R-loop removal is linked to changes in CTD modification, we used the 8WG16 antibody, which recognizes non-phosphorylated Ser2 residues and shows minimal enrichment at PcG-repressed genes (Stock et al., 2007; Brookes and Pombo, 2009). Interestingly, loss of R-loops after RNase H1 overexpression led to an increase of 8WG16 Pol II levels at PcG target genes (Figure 4A). However, *β-actin* showed decreased 8WG16 Pol II levels upon R-loop removal, consistent with the reduction in nascent and processed transcripts shown in Figures 1B and 1C. 8WG16 Pol II levels over the non-R-loop-forming *CyclinB1* gene remained unaffected. These results suggest that removal of R-loops at PcG target genes leads to a specific change in Pol II CTD modification that is now recognized by the 8WG16 antibody, an event that has been reported after loss of RING1B and H2Aub1 (Stock et al., 2007).

Since CTD modifications affect the detection of Pol II by 8WG16 epitope (Xie et al., 2006; Stock et al., 2007; Brookes and Pombo, 2009), we examined additional CTD modifications in these conditions. PcG targets genes also exhibited an increase in the Ser7P Pol II levels (Figure 4B), which marks active genes, in line with gene derepression observed upon loss of R-loops (Figures 1B, 1C, and 4A). Again, at the promoter of *β-actin*, but not *CyclinB1*, depletion of R-loops led to a decrease in Ser7P Pol II levels (Figure 4B, right panel).

R-loop depletion had no detectable effect on Ser5P occupancy levels (Figure 4C) over PcG target genes, implying that this CTD modification precedes R-loop formation and is not affected by decreased occupancy of PcG enzymes upon R-loop depletion. These data suggest that R-loops contribute to the transcriptional repression of PcG target genes via changes that affect not only PcG stability on chromatin but also Pol II activation.

Constitutive Loss of EZH2 Does Not Affect R-Loop Formation, RING1B Recruitment, or Repression of R-Loop-Positive PcG Genes

To investigate the role of PcG presence on R-loop formation and gene repression at PcG-repressed genes, we created constitutive knockout (KO) mESCs (parental mESC clone E14) for *Ezh2* by introducing three constitutive stop codons at the beginning of exon 7 using the CRISPR/Cas9 system. The loss of EZH2 protein levels and chromatin occupancy, as well as the loss of the H3K27me3 mark on chromatin, was confirmed over our model genes (Figures S6A–S6C). Residual H3K27me3 was detected possibly due to the presence of EZH1, a homolog of EZH2 that can complement its activity (Margueron et al., 2008; Shen et al., 2008).

To investigate whether R-loop formation is affected upon PRC2 and H3K27me3 loss, we performed DRIP assays. First, we confirmed R-loop presence over PcG-repressed genes in the matched wild-type (WT) mESC clone. Importantly, R-loops were unaffected in *Ezh2* KO cells over R-loop-forming PcG-repressed genes, and control gene *β-actin* also remained unaffected, as expected. The negative control genes *Pax3*, *Mogat1*, *Hoxa7*, and *CyclinB1* showed no or very little enrichment, as expected (Figure 5A). This result is supported by the presence of Pol II Ser5P in the same conditions (Figure S6D) and importantly reveals that R-loops form over PcG-repressed genes irrespectively of EZH2 presence.

We next tested whether KO of *Ezh2* causes transcriptional derepression of R-loop-positive PcG-repressed genes. Analysis of spliced transcripts revealed that R-loop-positive PcG-repressed

(C) Proportion of PcG-repressed, active, and inactive genes that overlap with R-loops. Percentage of genes in each group that overlap with an R-loop peak but do not overlap with another R-loop-positive peak (dark color) or that overlap with other R-loop-positive peaks (light color) are shown.
(D) GRO-seq RPKM data in R-loop-positive and negative Polycomb targets.
(E) Number of PcG-repressed genes giving rise to R-loops and nascent RNA (GRO-seq, RPKM > 0.1).
(F–I) DRIP (F), RNA analysis (G and H), and EZH2 ChIP (I) on *Pax3*, *Hoxa7*, and *Mogat1* genes (n = 3).
Error bars represent SD.

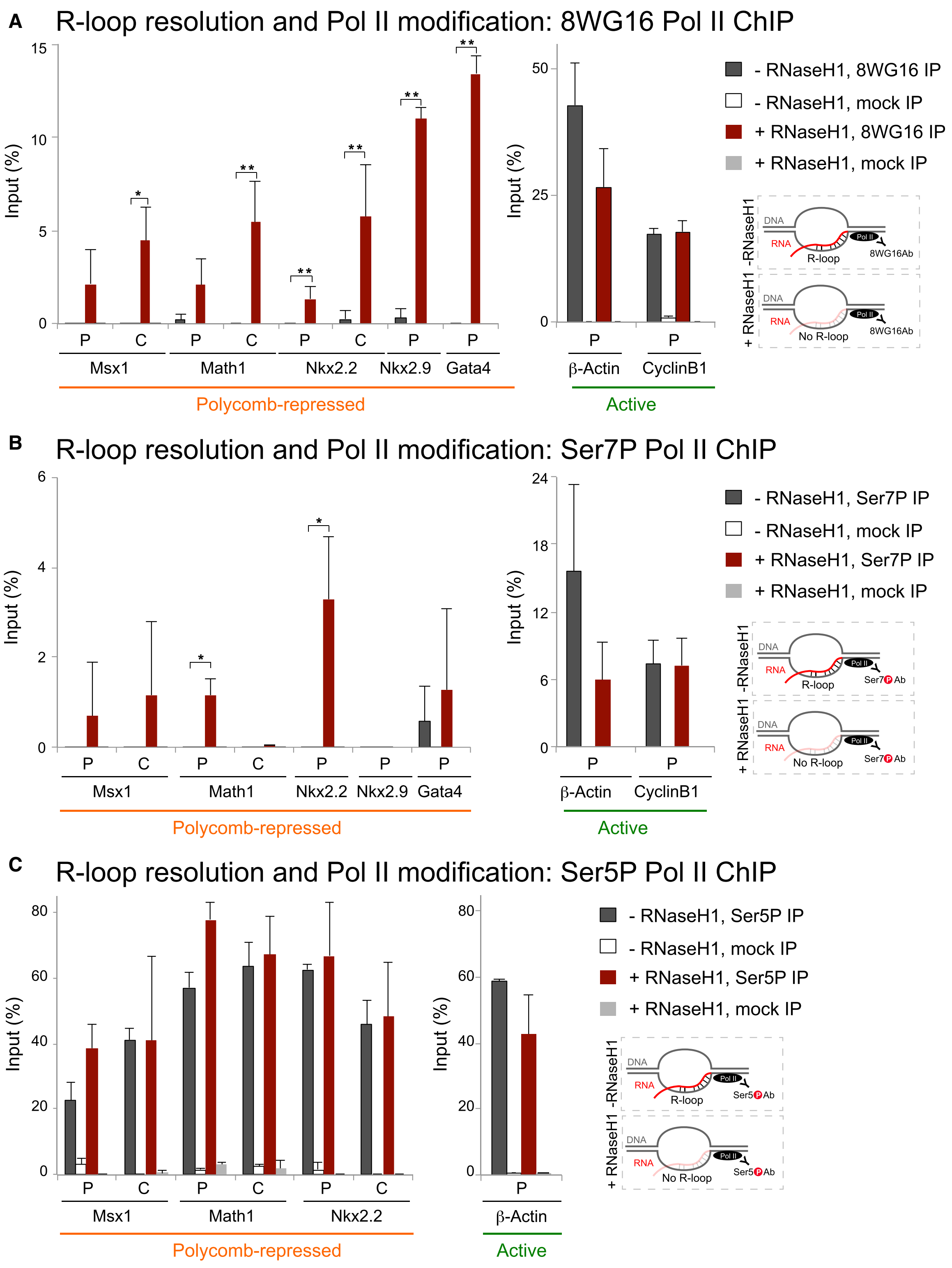

Figure 4. Selective Removal of R-Loops Leads to an Activation of Pol II State over PcG-Repressed Genes

(A–C) 8WG16 (A), Ser7P (B), and Ser5P (C) ChIP analyses upon RNase H1 overexpression on PcG-repressed and active genes. Error bars represent SD; n = 3. *p < 0.05, **p < 0.01, ***p < 0.001.

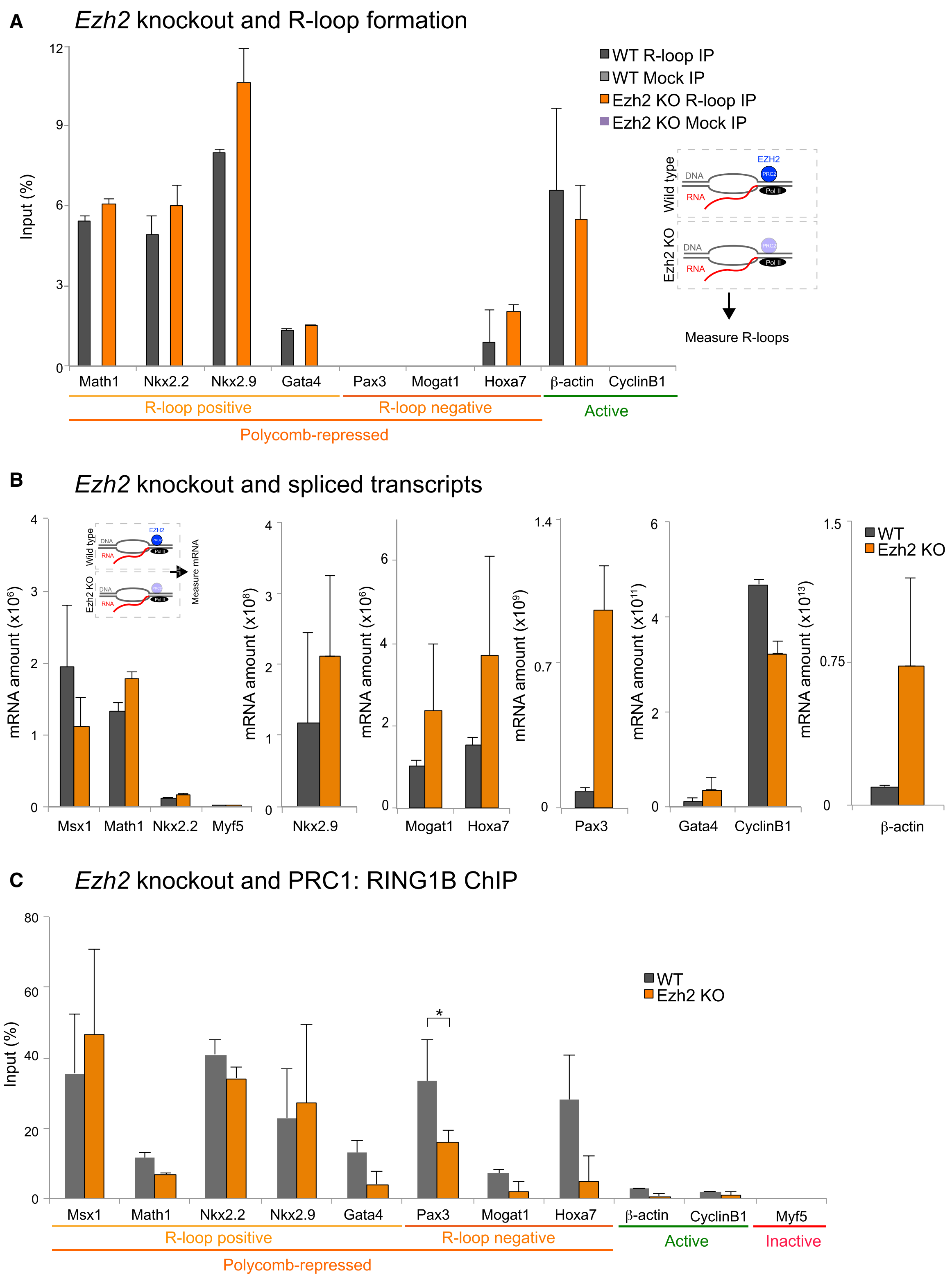

Figure 5. Constitutive Loss of EZH2 Does Not Affect R-Loops, Gene Repression, and RING1B Recruitment

(A) R-loop analysis in WT and *Ezh2* knockout (KO) mESCs.

(B) qRT-PCR analysis of total RNA on indicated genes in WT and *Ezh2* KO mESCs.

(C) RING1B Pol II ChIP analysis over PcG-repressed and active genes.

Error bars represent SD; n = 3. *p < 0.05, *p < 0.01, **p < 0.001.

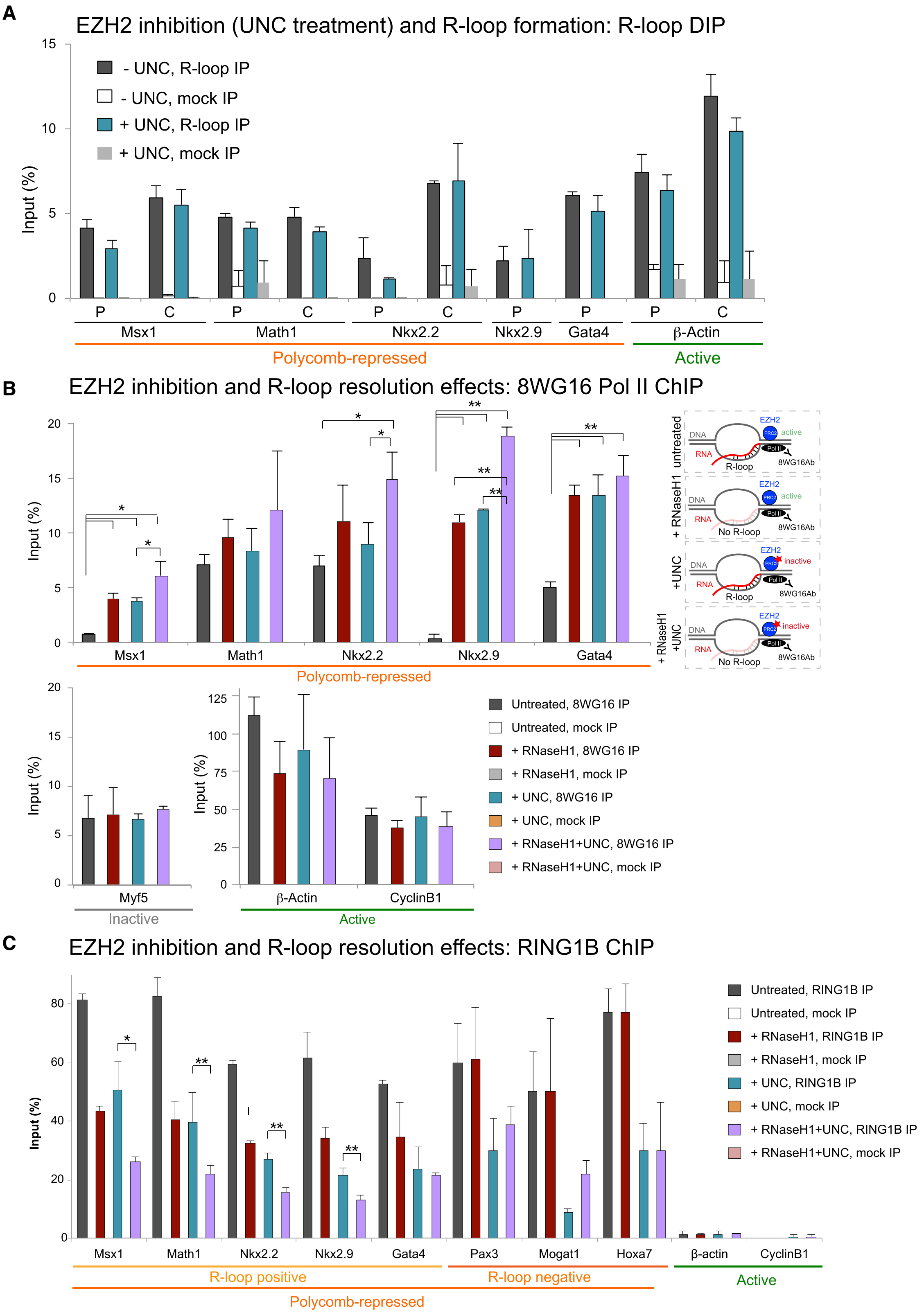

(legend on next page)

genes do not show prevalent upregulation of their mature transcripts upon *Ezh2* KO. Interestingly, R-loop-negative PcG-repressed genes exhibited slight (*Mogat1* and *Hoxa7*) or higher (*Pax3*) derepression upon constitutive *Ezh2* loss (Figure 5B). *β-actin* showed an increase on mature transcripts upon loss of *Ezh2*, in line with studies showing upregulation of highly expressed active genes in *Eed* KO mESC, due to increase in H3K27me1 levels in these conditions (Ferrari et al., 2014). Finally, 8WG16 Pol II levels were also unaffected over R-loop-positive PcG-repressed genes in *Ezh2* KO cells (Figure S6E), consistent with no evident increase in spliced transcript levels.

The maintained repression of R-loop-positive PcG target genes upon constitutive EZH2 loss prompted us to investigate how PRC1 recruitment is affected upon R-loop formation. Strikingly, ChIP analysis revealed that RING1B levels at R-loop-positive genes were largely unaffected in the absence of EZH2, despite the reduction in H3K27me3 (Figure 5D). However, RING1B levels in R-loop-negative genes were reduced in *Ezh2* KO cells, consistent with the mild transcriptional derepression observed in these conditions. These results collectively predict that R-loops and RING1B presence could account, synergistically or independently, for the lack of derepression specifically over R-loop-positive PcG-repressed genes in the absence of EZH2 and upon reduced H3K27me3.

Chemical Inhibition of EZH2 Causes Gene Derepression without Loss of R-Loops

The findings that R-loops, RING1B, and gene repression were maintained upon *Ezh2* KO prompted us to interfere with both EZH1 and EZH2 methyltransferase activity. We used UNC1999 (UNC), an inhibitor that prevents H3K27me3 deposition at PcG-repressed genes without disrupting EZH1 and EZH2 chromatin binding (Konze et al., 2013; Xu et al., 2015; Rizq et al., 2017).

Upon UNC treatment, H3K27me3 levels were reduced (Figure S7A), whereas EZH2 binding on chromatin remained unaffected (Figure S7B). Notably, R-loops remained unaffected upon UNC treatment (Figure 6A), strongly suggesting that R-loops form upstream of both EZH1-2 activity and presence (Figure 5A). Again, Ser5P Pol II occupancy was also maintained over R-loop-positive PcG-repressed genes (Figure S7C). UNC treatment and reduction of H3K27me3 led to derepression of R-loop-positive PcG target genes at the level of 8WG16 Pol II and spliced mRNA and was sufficient to deplete RING1B occupancy (Figures S7D–S7F).

R-Loops and RING1B Recruitment Both Contribute to the Transcriptional Repression of PcG Targets

We have showed that absence of EZH2 protein alone is not enough to alter the transcriptional status of R-loop-positive PcG-repressed genes, whereas interference either with R-loops or EZH1-2 catalytic activity causes their transcriptional derepression. To investigate whether R-loops and EZH2 activity act through parallel pathways or have synergistic effects on the silencing at PcG target genes, we combined R-loop removal and EZH2 inhibition and performed 8WG16 Pol II ChIP under the following conditions (Figure 6B): (1) untreated mock-transfected cells, where R-loops and EZH2 activity are both intact; (2) untreated cells overexpressing RNase H1, where R-loops are diminished and EZH2 and RING1B recruitment are reduced; (3) UNC-treated cells, where EZH2 still binds to chromatin but its catalytic activity is compromised and R-loops are still formed but RING1B is not recruited; and (4) UNC-treated cells overexpressing RNase H1, where both R-loops and EZH2 methyltransferase activity are deregulated. Remarkably, we observed a higher increase in 8WG16 Pol II over PcG-repressed genes in UNC-treated cells overexpressing RNase H1 (purple bars) than in cells with either R-loop depletion (red bars) or EZH2 inhibition (blue bars) alone, suggesting that both R-loops and EZH2 catalytic activity contribute to PcG repression. This effect was not observed at active genes (Figure 6B, bottom right panel).

We then tested whether the reduced recruitment of RING1B could account for the enhanced gene derepression observed in the combined conditions of R-loops loss and inhibition of PRC2 activity. After performing RING1B ChIP (Figure 6C) as above, we found reduced RING1B occupancy on chromatin upon R-loop resolution (red bars) and upon PRC2 inhibition (blue bars), as expected from our previous findings (Figures 2D and S7F). Importantly, we observed a further significant decrease in RING1B recruitment over PcG target genes that form R-loops when PRC2 inhibition was combined with R-loop resolution (RNaseH1 + UNC; purple bars), as compared to UNC treatment alone (blue bars). This effect was specific to R-loop-positive genes, as R-loop-negative PcG-repressed genes showed no depletion in RING1B occupancy in combined conditions of R-loop resolution and UNC treatment. These results indicate that both R-loop formation and RING1B recruitment on chromatin are important to repress a subset of PcG targets that form R-loops and, importantly, highlight that RING1B recruitment and occupancy on chromatin can be also regulated by R-loop formation in the absence of PRC2 activity.

R-Loops Inhibit Productive Gene Expression Independently of EZH2 Occupancy on Chromatin

We next sought to investigate whether EZH2 (PRC2) occupancy on chromatin is important for gene repression in R-loop-forming PcG targets. We went back to the *Ezh2* KO system and tested whether removal of R-loops could induce gene activation in the absence of EZH2. We performed 8WG16 Pol II ChIP as a proxy for gene activation in the following conditions: (1) WT mock-transfected cells (Figure 7A, gray bars), (2) WT cells overexpressing RNase H1 (red bars), (3) *Ezh2* KO cells (orange bars), and (4) *Ezh2* KO cells overexpressing RNase H1 (green bars). Removal

Figure 6. Catalytic Inhibition of EZH2 and Loss of R-loops Result in Enhanced Transcriptional Derepression and Reduced RING1B Recruitment

(A) R-loop analysis upon UNC treatment.

(B and C) 8WG16 (B) Pol II and RING1B (C) ChIP analyses on indicated genes with or without UNC treatment minus or plus RNase H1. (C) *$p < 0.05$, **$p < 0.01$, ***$p < 0.001$.

Error bars represent SD; n = 3.

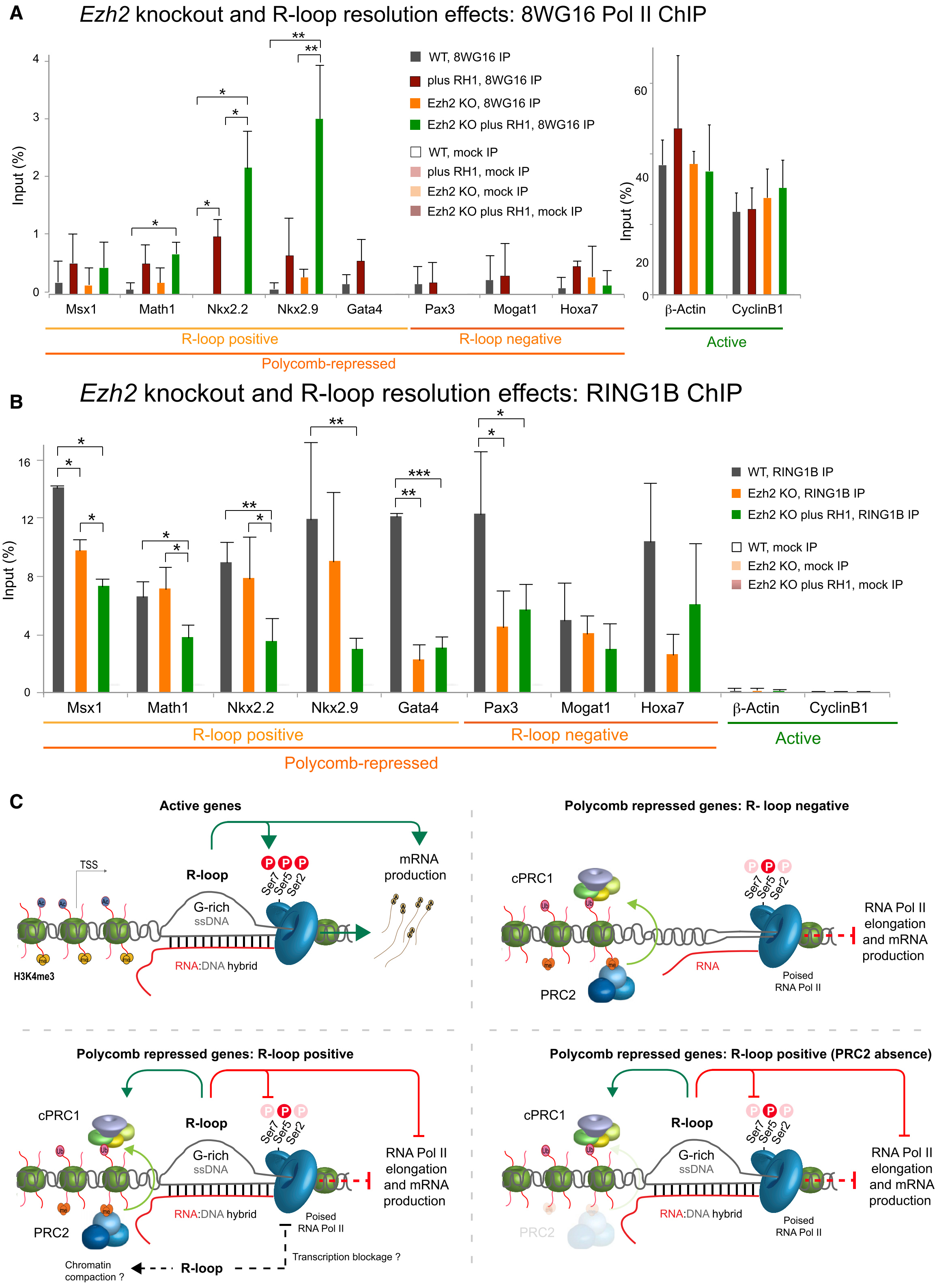

(legend on next page)

of R-loops led to an increase in 8WG16 Pol II levels in WT mESCs (Figure 7A, red bars). As observed before (Figure 5C), *Ezh2* KO cells do not exhibit signs of transcriptional activation. Interestingly, removal of R-loops in *Ezh2* KO cells leads to an increase in 8WG16 Pol II levels (green bars) over R-loop-positive PcG-repressed genes, indicative of gene activation. *Pax3*, *Mogat1*, and *Hoxa7*, PcG-repressed genes without R-loops, remained repressed. These results reveal that R-loop structures can act as transcriptional repressors in the PcG system independently of EZH2 (PRC2).

Given that in *Ezh2* KO conditions RING1B was unaffected at R-loop-positive genes (Figure 5) and that RING1B recruitment on chromatin can also be regulated by R-loops (Figure 6C), we wondered whether the transcriptional change in *Ezh2* KO cells without R-loops could be due to changes in RING1B occupancy. We performed RING1B ChIP in *Ezh2* KO cells overexpressing RNase H1 and used WT and *Ezh2* KO cells as controls (Figure 7B). Remarkably, R-loop resolution by RNase H1 overexpression (green bars) led to a decrease in RING1B levels in all R-loop-positive PcG-repressed genes as compared to *Ezh2* KO (orange bars) and WT (gray bars) cells. *Pax3*, *Mogat1*, and *Hoxa7* genes exhibited no change in RING1B occupancy upon R-loop resolution in *Ezh2* KO cells. The above results suggested that R-loop formation is important for RING1B recruitment specifically at the subset of PcG-repressed genes that form R-loops.

DISCUSSION

Our results have collectively uncovered several unanticipated aspects of PcG-repression mechanisms at developmental regulator genes in mESCs, which consist of a synergistic interplay between R-loops and RING1B, the catalytic subunit of PRC1. We have identified a novel repression mechanism of PcG targets where R-loops are sufficient to repress, independently of EZH2 and of H3K27me3 and H2Aub1 chromatin marks.

Experiments targeting the catalytic activity of EZH1-2 allowed us to further dissect the role of RING1B in PcG regulation. EZH2 chemical inhibition resulted in reduction of RING1B and transcriptional activation. These results are in line with previous studies showing that the canonical PRC1 can be recruited to chromatin via prior H3K27me3 deposition mediated by PRC2 (Wang et al., 2004; Boyer et al., 2006). We now show that in the absence of PRC2 activity, RING1B can be recruited over PcG targets via the formation of R-loops. Interestingly R-loop profiles remained unaffected in *Ezh2* KO and upon chemical inhibition of EZH2, suggesting that R-loop formation may be a primary step in the repression pathway of R-loop-positive PcG targets.

We show a relationship between R-loops and RING1B, which is an intriguing new aspect of PcG regulation. PRC2 has been reported to bind RNA (Davidovich et al., 2013; Kaneko et al., 2013; Davidovich et al., 2015; Beltran et al., 2016; Wang et al., 2017), as was the CBX7 subunit of PRC1 (Bernstein et al., 2006b; Yap et al., 2010; Pintacuda et al., 2017; Rosenberg et al., 2017). However, there is no evidence for RING1B binding to RNA-containing structures, such as R-loops. R-loops can act as transcriptional repressors by slowing down Pol II (Skourti-Stathaki et al., 2011) and causing transcriptional blockage *in vitro* (Belotserkovskii et al., 2010). It is therefore possible that R-loops orchestrate the initial signal of transcriptional repression sensed by PRC1. R-loops have also been linked to chromatin compaction (Santos-Pereira and Aguilera, 2015; Chédin, 2016) and could synergize with RING1B to compromise the elongation competence of Pol II (Francis et al., 2004; Eskeland et al., 2010; Endoh et al., 2012).

Our data reveal a novel class of PcG-repressed genes in mESCs (Figure 7C). At R-loop-positive PcG-repressed genes, R-loop formation interferes with the establishment of an elongating, active form of Pol II, which may initiate a defective state of gene expression. This effect can in turn assist RING1B to sense the transcriptional repression over these regions, synergize with EZH2, and actively impose silencing of PcG-repressed genes. In the absence of PRC2, R-loops can recruit or stabilize RING1B on chromatin and together impose transcriptional repression. Our results importantly highlight that PcG repression at developmental regulator genes in mESCs does not occur via a single mechanism but instead consists of different layers of repression, some of which are specific to the gene subset.

Previously, SUZ12 occupancy was shown to be increased upon loss of R-loops (Chen et al., 2015). However, in our experimental setting, we did not observe a significant effect. SUZ12 occupancy genome-wide is almost unchanged upon R-loop resolution, as opposed to the reduction observed in EZH2 occupancy in the same conditions. It therefore remains an open question how different subunits are recruited and stabilized at PcG-repressed genes.

Our experimental strategy also highlighted differences between R-loop function over PcG-repressed and active genes (Figure 7C). Even though R-loops form over both sets of genes in mESCs, their lifetimes are different, and they display opposite roles in gene regulation at these two different genomic contexts of the same cell type. R-loops over PcG targets are less sensitive to transcription inhibition with DRB than over active genes, suggesting that their stability can depend on the gene context. R-loops act as transcriptional repressors in PcG-repressed genes but as transcriptional activators in active genes, the latter confirming previous observations in different cell types. Features such as the quality and fate of the nascent RNA involved in R-loop formation or the stability and length of R-loops in different loci could account for this binary effect. Future studies on the regulation and function of R-loops as "activators" and "repressors" will shed light into this intriguing duality.

Altogether, the current evidence supports functional associations among DNA, transcription, and chromatin over PcG-

Figure 7. R-Loops and RING1B Recruitment Contribute to the Transcriptional Repression of PcG Targets in the Absence of EZH2

(A) 8WG16 Pol II ChIP analysis on indicated genes in WT or *Ezh2* KO cells minus or plus RNase H1.

(B) RING1B ChIP analysis in *Ezh2* KO cells minus or plus RNase H1.

In (A) and (B), error bars represent SD; n = 3. $^{*}p < 0.05$, $^{**}p < 0.01$, $^{***}p < 0.001$.

(C) The role of R-loops in the transcriptional repression of R-loop-positive PcG targets. The model is explained in the text. cPRC1, canonical PRC1.

repressed genes. We now establish that this interplay can be regulated by the formation of R-loops and that RING1B may play a vital role in this pathway for transcriptional repression. These results provide a conceptual advance in our understanding of R-loop biology and PcG regulation.

STAR★METHODS

Detailed methods are provided in the online version of this paper and include the following:

- KEY RESOURCES TABLE
- CONTACT FOR REAGENT AND RESOURCE SHARING
- EXPERIMENTAL MODEL AND SUBJECT DETAILS
- METHOD DETAILS
  - Cell treatments
  - Generation of Ezh2 KO cell line
  - DIP/DRIP analysis
  - RNA analysis
  - Cross-linked ChIP analysis
  - ChIP-sequencing
  - Native ChIP analysis
  - Sequential native ChIP analysis
  - Western blot analysis
- QUANTIFICATION AND STATISTICAL ANALYSIS
  - Bio-informatic analyses
  - R-loop genome-wide analysis
  - p values and Statistical analysis
- DATA AND SOFTWARE AVAILABILITY

SUPPLEMENTAL INFORMATION

Supplemental Information includes seven figures and one table and can be found with this article online at https://doi.org/10.1016/j.molcel.2018.12.016.

ACKNOWLEDGMENTS

This work was supported by a Sir Henry Wellcome Fellowship (grant 101489/Z/13/Z to K.S.-S.), the Wellcome Trust (grant 091580/Z/10/Z and Investigator Award 107930/Z/15/Z to A.B.), a Sir Henry Dale Fellowship (104175/Z/14/Z to P.V.), and funding from the European Research Council (ERC) under the European Union's Horizon 2020 research and innovation programme (ERC-STG grant agreement 639253 to P.V.). The Wellcome Centre for Cell Biology is supported by core funding from the Wellcome Trust (203149). A.P. and E.T.T. thank the Helmholtz Association (11431). We thank Tiago Rito (Pombo lab, currently Rockefeller University, USA) for initial processing of GRO-seq data, R.J. Crouch (NIH, Bethesda, MD, USA) for the GFP-RNase H1 plasmid, João Dias (Pombo lab) for advice on UNC treatment, Elana Bryan and Kim Webb (Voigt lab) for help generating the *Ezh2* KO cell line, and Ricardo Nunes Bastos (Oxford Nanoimaging, UK) for help with figure formatting. We finally thank the members of A.P. and A.B. labs for advice and fruitful discussions.

AUTHOR CONTRIBUTIONS

K.S.-S. conceived the study and designed and performed all the molecular biology experiments. M.W. and P.V. generated, characterized, and provided the *Ezh2* KO cell line. E.T.T. performed the bioinformatics analyses, supervised by A.P. K.S.-S. discussed and interpreted results with input from A.P., E.T.T., P.V., and A.B. K.S.-S. wrote the manuscript with input from A.P. and E.T.T., and all authors revised it.

DECLARATION OF INTERESTS

The authors declare no competing interests.

Received: December 11, 2017
Revised: September 14, 2018
Accepted: December 14, 2018
Published: January 29, 2019

REFERENCES

Aguilera, A., and García-Muse, T. (2012). R loops: from transcription byproducts to threats to genome stability. Mol. Cell *46*, 115–124.

Azuara, V., Perry, P., Sauer, S., Spivakov, M., Jørgensen, H.F., John, R.M., Gouti, M., Casanova, M., Warnes, G., Merkenschlager, M., and Fisher, A.G. (2006). Chromatin signatures of pluripotent cell lines. Nat. Cell Biol. *8*, 532–538.

Belotserkovskii, B.P., Liu, R., Tornaletti, S., Krasilnikova, M.M., Mirkin, S.M., and Hanawalt, P.C. (2010). Mechanisms and implications of transcription blockage by guanine-rich DNA sequences. Proc. Natl. Acad. Sci. USA *107*, 12816–12821.

Beltran, M., Yates, C.M., Skalska, L., Dawson, M., Reis, F.P., Viiri, K., Fisher, C.L., Sibley, C.R., Foster, B.M., Bartke, T., et al. (2016). The interaction of PRC2 with RNA or chromatin is mutually antagonistic. Genome Res. *26*, 896–907.

Bernstein, B.E., Mikkelsen, T.S., Xie, X., Kamal, M., Huebert, D.J., Cuff, J., Fry, B., Meissner, A., Wernig, M., Plath, K., et al. (2006a). A bivalent chromatin structure marks key developmental genes in embryonic stem cells. Cell *125*, 315–326.

Bernstein, E., Duncan, E.M., Masui, O., Gil, J., Heard, E., and Allis, C.D. (2006b). Mouse polycomb proteins bind differentially to methylated histone H3 and RNA and are enriched in facultative heterochromatin. Mol. Cell. Biol. *26*, 2560–2569.

Blackledge, N.P., Farcas, A.M., Kondo, T., King, H.W., McGouran, J.F., Hanssen, L.L.P., Ito, S., Cooper, S., Kondo, K., Koseki, Y., et al. (2014). Variant PRC1 complex-dependent H2A ubiquitylation drives PRC2 recruitment and polycomb domain formation. Cell *157*, 1445–1459.

Blackledge, N.P., Rose, N.R., and Klose, R.J. (2015). Targeting Polycomb systems to regulate gene expression: modifications to a complex story. Nat. Rev. Mol. Cell Biol. *16*, 643–649.

Boguslawski, S.J., Smith, D.E., Michalak, M.A., Mickelson, K.E., Yehle, C.O., Patterson, W.L., and Carrico, R.J. (1986). Characterization of monoclonal antibody to DNA.RNA and its application to immunodetection of hybrids. J. Immunol. Methods *89*, 123–130.

Boyer, L.A., Plath, K., Zeitlinger, J., Brambrink, T., Medeiros, L.A., Lee, T.I., Levine, S.S., Wernig, M., Tajonar, A., Ray, M.K., et al. (2006). Polycomb complexes repress developmental regulators in murine embryonic stem cells. Nature *441*, 349–353.

Breiling, A., Turner, B.M., Bianchi, M.E., and Orlando, V. (2001). General transcription factors bind promoters repressed by Polycomb group proteins. Nature *412*, 651–655.

Brookes, E., and Pombo, A. (2009). Modifications of RNA polymerase II are pivotal in regulating gene expression states. EMBO Rep. *10*, 1213–1219.

Brookes, E., de Santiago, I., Hebenstreit, D., Morris, K.J., Carroll, T., Xie, S.Q., Stock, J.K., Heidemann, M., Eick, D., Nozaki, N., et al. (2012). Polycomb associates genome-wide with a specific RNA polymerase II variant, and regulates metabolic genes in ESCs. Cell Stem Cell *10*, 157–170.

Castellano-Pozo, M., Santos-Pereira, J.M., Rondón, A.G., Barroso, S., Andújar, E., Pérez-Alegre, M., García-Muse, T., and Aguilera, A. (2013). R loops are linked to histone H3 S10 phosphorylation and chromatin condensation. Mol. Cell *52*, 583–590.

Cerritelli, S.M., Frolova, E.G., Feng, C., Grinberg, A., Love, P.E., and Crouch, R.J. (2003). Failure to produce mitochondrial DNA results in embryonic lethality in Rnaseh1 null mice. Mol. Cell *11*, 807–815.

Chédin, F. (2016). Nascent connections: R-loops and chromatin patterning. Trends Genet. *32*, 828–838.

Chen, P.B., Chen, H.V., Acharya, D., Rando, O.J., and Fazzio, T.G. (2015). R loops regulate promoter-proximal chromatin architecture and cellular differentiation. Nat. Struct. Mol. Biol. *22*, 999–1007.

Chopra, V.S., Hong, J.-W., and Levine, M. (2009). Regulation of Hox gene activity by transcriptional elongation in Drosophila. Curr. Biol. *19*, 688–693.

Cooper, S., Dienstbier, M., Hassan, R., Schermelleh, L., Sharif, J., Blackledge, N.P., De Marco, V., Elderkin, S., Koseki, H., Klose, R., et al. (2014). Targeting polycomb to pericentric heterochromatin in embryonic stem cells reveals a role for H2AK119u1 in PRC2 recruitment. Cell Rep. *7*, 1456–1470.

Davidovich, C., Zheng, L., Goodrich, K.J., and Cech, T.R. (2013). Promiscuous RNA binding by Polycomb repressive complex 2. Nat. Struct. Mol. Biol. *20*, 1250–1257.

Davidovich, C., Wang, X., Cifuentes-Rojas, C., Goodrich, K.J., Gooding, A.R., Lee, J.T., and Cech, T.R. (2015). Toward a consensus on the binding specificity and promiscuity of PRC2 for RNA. Mol. Cell *57*, 552–558.

Dellino, G.I., Schwartz, Y.B., Farkas, G., McCabe, D., Elgin, S.C.R., and Pirrotta, V. (2004). Polycomb silencing blocks transcription initiation. Mol. Cell *13*, 887–893.

Di Croce, L., and Helin, K. (2013). Transcriptional regulation by Polycomb group proteins. Nat. Struct. Mol. Biol. *20*, 1147–1155.

Dias, J.D., Rito, T., Torlai Triglia, E., Kukalev, A., Ferrai, C., Chotalia, M., Brookes, E., Kimura, H., and Pombo, A. (2015). Methylation of RNA polymerase II non-consensus Lysine residues marks early transcription in mammalian cells. eLife *4*, e11215.

Egan, B., Yuan, C.-C., Craske, M.L., Labhart, P., Guler, G.D., Arnott, D., Maile, T.M., Busby, J., Henry, C., Kelly, T.K., et al. (2016). An alternative approach to ChIP-seq normalization enables detection of genome-wide changes in histone H3 lysine 27 trimethylation upon EZH2 inhibition. PLoS ONE *11*, e0166438.

Endoh, M., Endo, T.A., Endoh, T., Isono, K., Sharif, J., Ohara, O., Toyoda, T., Ito, T., Eskeland, R., Bickmore, W.A., et al. (2012). Histone H2A mono-ubiquitination is a crucial step to mediate PRC1-dependent repression of developmental genes to maintain ES cell identity. PLoS Genet. *8*, e1002774.

Eskeland, R., Leeb, M., Grimes, G.R., Kress, C., Boyle, S., Sproul, D., Gilbert, N., Fan, Y., Skoultchi, A.I., Wutz, A., and Bickmore, W.A. (2010). Ring1B compacts chromatin structure and represses gene expression independent of histone ubiquitination. Mol. Cell *38*, 452–464.

Farcas, A.M., Blackledge, N.P., Sudbery, I., Long, H.K., McGouran, J.F., Rose, N.R., Lee, S., Sims, D., Cerase, A., Sheahan, T.W., et al. (2012). KDM2B links the Polycomb repressive complex 1 (PRC1) to recognition of CpG islands. eLife *1*, e00205.

Ferrai, C., Torlai Triglia, E., Risner-Janiczek, J.R., Rito, T., Rackham, O.J., de Santiago, I., Kukalev, A., Nicodemi, M., Akalin, A., Li, M., et al. (2017). RNA polymerase II primes Polycomb-repressed developmental genes throughout terminal neuronal differentiation. Mol. Syst. Biol. *13*, 946.

Ferrari, P., and Strubin, M. (2015). Uncoupling histone turnover from transcription-associated histone H3 modifications. Nucleic Acids Res. *43*, 3972–3985.

Ferrari, K.J., Scelfo, A., Jammula, S., Cuomo, A., Barozzi, I., Stützer, A., Fischle, W., Bonaldi, T., and Pasini, D. (2014). Polycomb-dependent H3K27me1 and H3K27me2 regulate active transcription and enhancer fidelity. Mol. Cell *53*, 49–62.

Francis, N.J., Kingston, R.E., and Woodcock, C.L. (2004). Chromatin compaction by a polycomb group protein complex. Science *306*, 1574–1577.

Ginno, P.A., Lott, P.L., Christensen, H.C., Korf, I., and Chédin, F. (2012). R-loop formation is a distinctive characteristic of unmethylated human CpG island promoters. Mol. Cell *45*, 814–825.

Ginno, P.A., Lim, Y.W., Lott, P.L., Korf, I., and Chédin, F. (2013). GC skew at the 5′ and 3′ ends of human genes links R-loop formation to epigenetic regulation and transcription termination. Genome Res. *23*, 1590–1600.

Guenther, M.G., Levine, S.S., Boyer, L.A., Jaenisch, R., and Young, R.A. (2007). A chromatin landmark and transcription initiation at most promoters in human cells. Cell *130*, 77–88.

Hamperl, S., and Cimprich, K.A. (2014). The contribution of co-transcriptional RNA:DNA hybrid structures to DNA damage and genome instability. DNA Repair (Amst.) *19*, 84–94.

Harlen, K.M., and Churchman, L.S. (2017). The code and beyond: transcription regulation by the RNA polymerase II carboxy-terminal domain. Nat. Rev. Mol. Cell Biol. *18*, 263–273.

He, J., Shen, L., Wan, M., Taranova, O., Wu, H., and Zhang, Y. (2013). Kdm2b maintains murine embryonic stem cell status by recruiting PRC1 complex to CpG islands of developmental genes. Nat. Cell Biol. *15*, 373–384.

Hsin, J.-P., and Manley, J.L. (2012). The RNA polymerase II CTD coordinates transcription and RNA processing. Genes Dev. *26*, 2119–2137.

Jonkers, I., Kwak, H., and Lis, J.T. (2014). Genome-wide dynamics of Pol II elongation and its interplay with promoter proximal pausing, chromatin, and exons. eLife *3*, e02407.

Kaneko, S., Son, J., Shen, S.S., Reinberg, D., and Bonasio, R. (2013). PRC2 binds active promoters and contacts nascent RNAs in embryonic stem cells. Nat. Struct. Mol. Biol. *20*, 1258–1264.

Kanhere, A., Viiri, K., Araújo, C.C., Rasaiyaah, J., Bouwman, R.D., Whyte, W.A., Pereira, C.F., Brookes, E., Walker, K., Bell, G.W., et al. (2010). Short RNAs are transcribed from repressed polycomb target genes and interact with polycomb repressive complex-2. Mol. Cell *38*, 675–688.

Kim, D., Pertea, G., Trapnell, C., Pimentel, H., Kelley, R., and Salzberg, S.L. (2013). TopHat2: accurate alignment of transcriptomes in the presence of insertions, deletions and gene fusions. Genome Biology *14*, R36.

Konze, K.D., Ma, A., Li, F., Barsyte-Lovejoy, D., Parton, T., Macnevin, C.J., Liu, F., Gao, C., Huang, X.-P., Kuznetsova, E., et al. (2013). An orally bioavailable chemical probe of the lysine methyltransferases EZH2 and EZH1. ACS Chem. Biol. *8*, 1324–1334.

Kouskouti, A., and Talianidis, I. (2005). Histone modifications defining active genes persist after transcriptional and mitotic inactivation. EMBO J. *24*, 347–357.

Ku, M., Koche, R.P., Rheinbay, E., Mendenhall, E.M., Endoh, M., Mikkelsen, T.S., Presser, A., Nusbaum, C., Xie, X., Chi, A.S., et al. (2008). Genomewide analysis of PRC1 and PRC2 occupancy identifies two classes of bivalent domains. PLoS Genet. *4*, e1000242.

Langmead, B., and Salzberg, S.L. (2012). Fast gapped-read alignment with Bowtie 2. Nat. Methods *9*, 357–359.

Margueron, R., and Reinberg, D. (2011). The Polycomb complex PRC2 and its mark in life. Nature *469*, 343–349.

Margueron, R., Li, G., Sarma, K., Blais, A., Zavadil, J., Woodcock, C.L., Dynlacht, B.D., and Reinberg, D. (2008). Ezh1 and Ezh2 maintain repressive chromatin through different mechanisms. Mol. Cell *32*, 503–518.

Mikkelsen, T.S., Ku, M., Jaffe, D.B., Issac, B., Lieberman, E., Giannoukos, G., Alvarez, P., Brockman, W., Kim, T.-K., Koche, R.P., et al. (2007). Genome-wide maps of chromatin state in pluripotent and lineage-committed cells. Nature *448*, 553–560.

Nakama, M., Kawakami, K., Kajitani, T., Urano, T., and Murakami, Y. (2012). DNA-RNA hybrid formation mediates RNAi-directed heterochromatin formation. Genes Cells *17*, 218–233.

Pintacuda, G., Wei, G., Roustan, C., Kirmizitas, B.A., Solcan, N., Cerase, A., Castello, A., Mohammed, S., Moindrot, B., Nesterova, T.B., and Brockdorff, N. (2017). hnRNPK recruits PCGF3/5-PRC1 to the Xist RNA B-repeat to establish Polycomb-mediated chromosomal silencing. Mol. Cell *68*, 955–969.e10.

Powell, W.T., Coulson, R.L., Gonzales, M.L., Crary, F.K., Wong, S.S., Adams, S., Ach, R.A., Tsang, P., Yamada, N.A., Yasui, D.H., et al. (2013). R-loop formation at Snord116 mediates topotecan inhibition of Ube3a-antisense and allele-specific chromatin decondensation. Proc. Natl. Acad. Sci. USA *110*, 13938–13943.

Ran, F.A., Hsu, P.D., Wright, J., Agarwala, V., Scott, D.A., and Zhang, F. (2013). Genome engineering using the CRISPR-Cas9 system. Nat. Protoc. *8*, 2281–2308.

Riising, E.M., Comet, I., Leblanc, B., Wu, X., Johansen, J.V., and Helin, K. (2014). Gene silencing triggers polycomb repressive complex 2 recruitment to CpG islands genome wide. Mol. Cell *55*, 347–360.

Rizq, O., Mimura, N., Oshima, M., Saraya, A., Koide, S., Kato, Y., Aoyama, K., Nakajima-Takagi, Y., Wang, C., Chiba, T., et al. (2017). Dual inhibition of EZH2 and EZH1 sensitizes PRC2-dependent tumors to proteasome inhibition. Clin. Cancer Res. *23*, 4817–4830.

Rosenberg, M., Blum, R., Kesner, B., Maier, V.K., Szanto, A., and Lee, J.T. (2017). Denaturing CLIP, dCLIP, pipeline identifies discrete RNA footprints on chromatin-associated proteins and reveals that CBX7 targets 3′ UTRs to regulate mRNA expression. Cell Syst. *5*, 368–385.e15.

Santos-Pereira, J.M., and Aguilera, A. (2015). R loops: new modulators of genome dynamics and function. Nat. Rev. Genet. *16*, 583–597.

Sanz, L.A., Hartono, S.R., Lim, Y.W., Steyaert, S., Rajpurkar, A., Ginno, P.A., Xu, X., and Chédin, F. (2016). Prevalent, dynamic, and conserved R-loop structures associate with specific epigenomic signatures in mammals. Mol. Cell *63*, 167–178.

Shen, X., Liu, Y., Hsu, Y.-J., Fujiwara, Y., Kim, J., Mao, X., Yuan, G.-C., and Orkin, S.H. (2008). EZH1 mediates methylation on histone H3 lysine 27 and complements EZH2 in maintaining stem cell identity and executing pluripotency. Mol. Cell *32*, 491–502.

Skourti-Stathaki, K., and Proudfoot, N.J. (2014). A double-edged sword: R loops as threats to genome integrity and powerful regulators of gene expression. Genes Dev. *28*, 1384–1396.

Skourti-Stathaki, K., Proudfoot, N.J., and Gromak, N. (2011). Human senataxin resolves RNA/DNA hybrids formed at transcriptional pause sites to promote Xrn2-dependent termination. Mol. Cell *42*, 794–805.

Skourti-Stathaki, K., Kamieniarz-Gdula, K., and Proudfoot, N.J. (2014). R-loops induce repressive chromatin marks over mammalian gene terminators. Nature *516*, 436–439.

Stock, J.K., Giadrossi, S., Casanova, M., Brookes, E., Vidal, M., Koseki, H., Brockdorff, N., Fisher, A.G., and Pombo, A. (2007). Ring1-mediated ubiquitination of H2A restrains poised RNA polymerase II at bivalent genes in mouse ES cells. Nat. Cell Biol. *9*, 1428–1435.

Sun, Q., Csorba, T., Skourti-Stathaki, K., Proudfoot, N.J., and Dean, C. (2013). R-loop stabilization represses antisense transcription at the Arabidopsis FLC locus. Science *340*, 619–621.

Tee, W.-W., Shen, S.S., Oksuz, O., Narendra, V., and Reinberg, D. (2014). Erk1/2 activity promotes chromatin features and RNAPII phosphorylation at developmental promoters in mouse ESCs. Cell *156*, 678–690.

Voigt, P., LeRoy, G., Drury, W.J., 3rd, Zee, B.M., Son, J., Beck, D.B., Young, N.L., Garcia, B.A., and Reinberg, D. (2012). Asymmetrically modified nucleosomes. Cell *151*, 181–193.

Wang, H., Wang, L., Erdjument-Bromage, H., Vidal, M., Tempst, P., Jones, R.S., and Zhang, Y. (2004). Role of histone H2A ubiquitination in Polycomb silencing. Nature *431*, 873–878.

Wang, X., Goodrich, K.J., Gooding, A.R., Naeem, H., Archer, S., Paucek, R.D., Youmans, D.T., Cech, T.R., and Davidovich, C. (2017). Targeting of Polycomb repressive complex 2 to RNA by short repeats of consecutive guanines. Mol. Cell *65*, 1056–1067.e5.

Xie, S.Q., Martin, S., Guillot, P.V., Bentley, D.L., and Pombo, A. (2006). Splicing speckles are not reservoirs of RNA polymerase II, but contain an inactive form, phosphorylated on serine2 residues of the C-terminal domain. Mol. Biol. Cell *17*, 1723–1733.

Xu, B., Konze, K.D., Jin, J., and Wang, G.G. (2015). Targeting EZH2 and PRC2 dependence as novel anticancer therapy. Exp. Hematol. *43*, 698–712.

Yap, K.L., Li, S., Muñoz-Cabello, A.M., Raguz, S., Zeng, L., Mujtaba, S., Gil, J., Walsh, M.J., and Zhou, M.-M. (2010). Molecular interplay of the noncoding RNA ANRIL and methylated histone H3 lysine 27 by polycomb CBX7 in transcriptional silencing of INK4a. Mol. Cell *38*, 662–674.

STAR★METHODS

KEY RESOURCES TABLE

REAGENT or RESOURCE	SOURCE	IDENTIFIER
Antibodies		
Mouse monoclonal anti-RNA-DNA hybrids S9.6	Hybridoma	N/A
Rabbit polyclonal anti-EZH2	Diagenode	pAb-039-050
Rabbit monoclonal anti-RING1B (clone D22F2)	Cell Signaling Technology	5694; RRID: AB_10705604
Rabbit polyclonal anti-SUZ12	Bethyl Laboratories	A302-407A; RRID: AB_1907290
Rabbit polyclonal anti-H3K27me3	Merck Millipore	07-449; RRID: AB_310624
Rabbit monoclonal anti-H2Aub1(clone D27C40)	Cell Signaling Technology	8240; RRID: AB_10891618
Mouse monoclonal anti-8WG16	BioLegend	920102; RRID: AB_2565318
Mouse monoclonal anti-Ser5P (clone CTD4H8)	Merck Millipore	05-623; RRID: AB_309852
Mouse monoclonal anti-Ser7P (clone 4E12)	Active Motif	61087; RRID: AB_2687452
Rabbit polyclonal anti-OCT4	Abcam	ab19857; RRID: AB_445175
Rabbit polyclonal anti-NANOG	Abcam	ab21624; RRID: AB_446437
Mouse monoclonal γ-tubulin (clone GTU-88)	Sigma-Aldrich	T5326; RRID: AB_532292
Chemicals, Peptides, and Recombinant Proteins		
UNC1999 inhibitor	Sigma-Aldrich	SML0778
5,6-Dichloro-1-β-D-ribofuranosylbenzimidazole (DRB)	Sigma-Aldrich	D1916
Critical Commercial Assays		
Chromatrap kit for Native ChIP	Chromatrap	500238
Multiplex Oligos for Illumina	New England Biolabs	E7335S and E7500S
RNA XP beads for library purification	Beckman Coulter	A63987
High Sensitivity DNA analysis kit	Agilent	5067-4626
NEBNext Ultra II DNA library kit for Illumina	New England Biolabs	E7645S
SuperScript III First-Strand Synthesis System	Thermo Fisher Scientific	18080051
Deposited Data		
Raw sequencing data EZH2 and SUZ12 −/+over-RNase H1 ChIP-seq	This study	GEO: GSE118115
Re-analyzed DRIP-seq data	Sanz et al., 2016	GEO: GSM1720620
Re-analyzed GRO-seq data	Jonkers et al., 2014	GEO: GSE48895
Re-analyzed H3K27me3 ChIP-seq data	Mikkelsen et al., 2007	GEO: GSM307619
Re-analyzed H2Aub1 ChIP-seq data	Brookes et al., 2012	GEO: GSM850471
Re-analyzed EZH2 ChIP-seq data	Ku et al., 2008	GEO: GSM327668
Re-analyzed RING1B ChIP-seq data	Ku et al., 2008	GEO: GSM327669
Re-analyzed RNAPII-S5p ChIP-seq data	Brookes et al., 2012	GEO: GSM850467
Re-analyzed RNAPII-8WG16 ChIP-seq data	Brookes et al., 2012	GEO: GSM850469
Re-analyzed RNAPII-S7p ChIP-seq data	Brookes et al., 2012	GEO: GSM850468
Re-analyzed RNAPII-S2p ChIP-seq data	Brookes et al., 2012	GEO: GSM850470
Re-analyzed mRNA-seq data	Brookes et al., 2012	GEO: GSM850476
Raw image data (Western blots)	Mendeley	https://doi.org/10.17632/55f4vg9ww4.1
Experimental Models: Cell Lines		
mESC clone 46C (WT)	Pombo lab	N/A
mESC clone E14 (WT)	Voigt lab	N/A
mESC *Ezh2* KO	This study	N/A
Oligonucleotides		
See Table S1		N/A

(*Continued on next page*)

Continued		
REAGENT or RESOURCE	SOURCE	IDENTIFIER
Software and Algorithms		
Bowtie v.2.0.5	Langmead and Salzberg, 2012	N/A
TopHat v.2.0.8	Kim et al., 2013	N/A
R	https://www.r-project.org	N/A

CONTACT FOR REAGENT AND RESOURCE SHARING

Further information and requests for resources and reagents should be directed to and will be fulfilled by the Lead Contact, Konstantina Skourti-Stathaki (kskourti@staffmail.ed.ac.uk).

EXPERIMENTAL MODEL AND SUBJECT DETAILS

Mouse ESC cells (46C, E14 and *Ezh2* KO) were grown on 0.1% gelatin-coated surfaces in GMEM BHK21 supplemented with 10% Fetal Calf Serum (FCS), 2mM L-glutamine, 1% MEM non-essential amino acids (NEAA), 1mM sodium pyruvate (GIBCO, Invitrogen), 50 μM 2-mercaptoethanol, 100 U/ml of human recombinant leukemia inhibitory factor (LIF, Chemicon, Millipore).

METHOD DETAILS

Cell treatments

Transfections with the GFP-RNase H1 plasmid into 46C, E14 and *Ezh2* KO mESCs were carried out as described previously (Skourti-Stathaki et al., 2011, 2014). Cells were harvested 48 h post-transfection. Treatment with 3 μM of UNC-1999 inhibitor (Sigma) was maintained for 72 hr and performed as described previously (Konze et al., 2013; Xu et al., 2015; Rizq et al., 2017). Control cells were treated with DMSO. Treatment with 80 μM of DRB inhibitor (Sigma) was performed as previously described (Sanz et al., 2016).

Generation of Ezh2 KO cell line

E14 mESCs were transfected with pX458 plasmid (Ran et al., 2013) encoding a guide RNA targeting exon 7 of the mouse *Ezh2* gene (20-bp target sequence CAGCAGGAAATTTCCGAGGT), along with a single-stranded DNA oligonucleotide for homology-directed repair to introduce three consecutive stop codons at the 5′ end of exon 7 (resulting sequence ATtAAtAAgCTTGatCACCTC, mutated bases in lower case). After fluorescence-based sorting for GFP-positive transfected single cells, single cell colonies were expanded, genotyped, and analyzed for EZH2 expression by western blot. Correct genotypes were confirmed by Sanger sequencing on PCR-amplified genomic material.

DIP/DRIP analysis

DNA immuno-precipitation (DIP/DRIP) analysis was carried out largely as described previously (Skourti-Stathaki et al., 2011) and was based on cross-linked ChIP analysis (see below) with some modifications. In essence, DIP analysis was performed without a cross-linking step, following the ChIP protocol with some modifications. After the nuclear lysis reaction, extracts were incubated with 30 μg of proteinase K (Roche) at 55°C genomic DNA was isolated. Following sonication, DIP analysis was carried out using antibody, recognizing RNA-DNA hybrids, purified from S9.6 hybridoma cell lines (Boguslawski et al., 1986). Washes and elution were carried out as in conventional ChIP analysis (see below). The immuno-precipitated, non-precipitated, and input DNAs were used as templates for qPCR. The PCR mixture contained QuantiTect SYBR green PCR master mix, 2 μL of the template DNA and primers from the Table S1. Final concentrations are shown as a % of the input value.

RNA analysis

Cells were washed with PBS and were harvested by adding 1 mL of Trizol reagent (ThermoFisher Scientific). RNA was then isolated following the manufacturer's instructions, DNase I treated for a total of 2 h (turbo DNA-*free*, Ambion kit), and reverse transcribed with SuperScript III Reverse Transcriptase (ThermoFisher Scientific), either using oligo-dT primer or a gene-specific primer from the Table S1, following the manufacturers' instructions.

Cross-linked ChIP analysis

Cells were fixed for 15 mins by addition of 36.5% methanol-stabilized formaldehyde solution, cross-linking was quenched by adding 1.32 mL of 1 M glycine and cells were washed and harvested in ice-cold PBS. Cells were lysed with cell lysis buffer (50mM Tris-HCl pH8, 2mM EDTA pH8, 0.1% NP40, 10% glycerol) and incubated for 10 mins on ice. The lysed cells were then centrifuged to pellet nuclei. The nuclear pellets were resuspended in Nuclear lysis buffer (1% SDS, 10mM EDTA, 50mM Tris-HCl pH8) and fragmented by sonication. 35-50 μg of chromatin was pre-cleared with A/G magnetic beads (ThermoFisher Scientific) for 1 h at 4°C and then

immuno-precipitated in IP dilution buffer (0.5%NP40, 200mM NaCl, 50mM Tris-HCl pH8) with 3.5-5 μg of antibody overnight. Washes were performed using low salt wash buffer (0.1% SDS, 0.5% NP40, 2mM EDTA, 20mM Tris-HCl pH8, 150mM NaCl), high salt wash buffer (0.1% SDS, 0.5% NP40, 2mM EDTA, 20mM Tris pH8, 500mM NaCl) and LiCl wash buffer (250mM LiCl, 0.5% NP40, 0.5% Na-deoxycholate, 1mM EDTA, 20mM Tris-HCl pH8) and eluted samples were reverse crosslinked for 4 hr to O/N hr at 65°C with 0.3 M NaCl and 3 μg/ml RNase A (Roche). Proteinase K treatment was performed for 2 hr at 45°C with 10 mM EDTA, 40 mM Tris-HCl pH 6.5 and 20 μg proteinase K. The chromatin was purified. The immuno-precipitated, non-precipitated, and input DNAs were used as templates for qPCR. The PCR mixture contained QuantiTect SYBR green PCR master mix, 2 μl of the template DNA and primers from the Table S1. Final concentrations are shown as a % of the input value.

The following antibodies were used for ChIP: anti-EZH2 (pAb-039-050, Diagenode), anti-RING1B (clone D22F2, 5694, Cell Signaling), anti-H3K27me3 (07-449, Millipore), anti-H2Aub1 (clone D27C4, 8240, Cell Signaling), anti-8WG16 (920102, Biolegend), anti-Ser5P (clone CTD4H8, 05-623, Millipore) and anti-Ser7P (clone 4E12, 61087, Active Motif).

ChIP-sequencing

ChIP protocol as described above was followed. The chromatin was purified using the MinElute PCR Purification kit (Cat. 28004, QIAGEN) and DNA concentration for library preparation was determined using Qubit fluorometric quantitation (ThermoFisher Scientific). Libraries were prepared from 8ng of DNA using the NEBNext Ultra II DNA library kit for Illumina (Cat. E7645S, NEB), following the manufacturers' instructions. Size selection was performed prior to PCR amplification using RNA clean XP beads (Cat. A63987, Beckman Coulter). Adaptors, PCR amplification and Index Primers were used to multiplex libraries (Multiplex oligos for Illlumina, Cat. E7335S and E7500S, NEB). Libraries were purified using RNA clean XP beads (Cat. A63987, Beckman Coulter) and library size was assessed before high-throughput sequencing by Bioanalyzer (Agilent) using the High Sensitivity DNA analysis kit (Cat. 5067-4626, Agilent). ChIP-seq libraries were sequenced paired-end on an Illumina HiSeq2500 sequencer at the Wellcome Trust Sanger Institute (Cambridge, UK). The following antibodies were used for ChIP-seq: anti-EZH2 (pAb-039-050, Diagenode) and anti-SUZ12 (A302-407A, Bethyl Laboratories).

Native ChIP analysis

Nascent ChIP analysis was carried out using the Chromatrap kit for native ChIP (Chromatrap) following the manufacturers' protocol. All buffers used were provided. In brief, non-crosslinked cells were harvested in ice-cold PBS and lysed in Hypotonic buffer. Nuclei were pelleted by nuclear separation. Chromatin was sheared using enzymatic shearing cocktail and smaller fragments were collected via centrifugation. Dialysis was performed O/N to remove unwanted contaminants and to obtain larger chromatin fragments. Small and large chromatin fragments were combined and immunoprecipitation was performed in a 35 μg:14 μg chromatin: antibody ratio, for all antibodies used in this study. Antibodies used were the same as indicated above.

Washes and elution was performed using the columns provided. Chromatin samples were digested with Proteinase K and DNA purification was performed. The immuno-precipitated, non-precipitated, and input DNAs were used as templates for qPCR. The PCR mixture contained QuantiTect SYBR green PCR master mix, 2 μl of the template DNA and primers from the Table S1. Final concentrations are shown as a % of the input value.

Sequential native ChIP analysis

Native chromatin was prepared and the first immunoprecipitation was performed as in single native ChIP using the Chromatrap kit (see above). After the elution of native chromatin in 50 μL total volume, the eluate was diluted 10-fold to obtain final concentration of 0.1% SDS for optimal second immunoprecipitation. The second immunoprecipitation, washes, and elution were then carried out following the single native ChIP protocol (see above). A no-antibody control was included in the second round of immunoprecipitation as a negative control, to test for contamination of antibody remaining from the first immunoprecipitation. All antibodies used are indicated above.

Western blot analysis

Cells were washed with ice-cold PBS. Cell pellets were resuspended in RIPA lysis buffer (50mM Tris pH7.5, 150mM NaCl, 150mM NP40, 0.5% Na-deoxycholate, 0.1% SDS, 10% glycerol). Cell lysis was performed for 20 mins on ice. Protein lysate was recovered by centrifugation and protein-containing supernatant was kept. Protein concentration was measured by Bradford assay (BIORAD). Western blotting was performed on 40 μg of total 46C mES cell protein extracts with antibodies raised against EZH2 (Diagenode), RING1B (Cell Signaling), OCT4 (Abcam), NANOG (Abcam) and γ-tubulin (Sigma), all at 1:1000 dilutions. Western blotting was performed with ECL kit (PerkinElmer).

QUANTIFICATION AND STATISTICAL ANALYSIS

Bio-informatic analyses

Mapping and processing of ChIP-seq datasets with Drosophila melanogaster *Spike-Ins*

ChIP-seq reads from paired-end sequencing (Illumina HiSeq 2500, 2x75bp) were aligned to the mouse genome mm9 and *Drosophila melanogaster* genome dm6 with Bowtie v2.0.5 (Langmead and Salzberg, 2012), with default parameters. Duplicated reads (i.e., identical

reads, aligned to the same genomic location) occurring more often than a threshold were removed. The threshold is computed for each dataset as the 95th percentile of the frequency distribution of reads.

To allow comparison between datasets, the amount of signal was normalized using *Drosophila* Spike-Ins as described in Active Motive catalog and as described in Egan et al. (2016). Briefly, the number of reads mapped to mouse was divided for the number of reads mapped to *Drosophila* in that dataset, then multiplied by 10^6 for convenience.

Average ChIP-seq profiles were generated as previously (Brookes et al., 2012), by plotting the average coverage in non-overlapping windows of 10 bp, across genomic windows centered on the TSS and the TES. Boxplots were produced using R.

R-loop genome-wide analysis

R-loop DRIP peaks in E14 ESCs from Sanz et al. (2016) were downloaded from GEO repository (GSM1720620). Gene list and classification were obtained from Brookes et al. (2012). Genes were classified as positive for R-loops if a R-loop peak overlapped the gene (defined as the genomic region 1kb before the gene's TSS to 1kb after the gene's TES). Positive genes that overlapped with other R-loop positive genes in the window described above were classified as 'uncertain due to proximity'.

Features of R-loop positive and R-loop negative PRC repressed genes

PRC repressed genes from Brookes et al. (2012) were divided into positive and negative for R-loops as described above. Most active genes (top 15%, n = 2829) or least active (bottom 15%, n = 2829) were defined as in Dias et al. (2015) using FPKM values, among genes negative for Polycomb marks (H3K27me3 and H2Aub1) from Brookes et al. (2012).

Nascent RNA RPKMs (reads per kilobase per million of reads mapped) were calculated based on the bedgraph files from Jonkers et al. (2014) (untreated ES cells downloaded from GEO: GSE48895). RPKM values represent the number of reads mapped in the sense of the gene from TSS to TES per kilobase (TSS to TES length) per million of reads mapped. Genes whose expression was >0.1 RPKM were considered positive for nascent RNA. As a technical note, GRO-seq relies on an *in vitro* transcription step by active transcribing polymerases which for Polycomb-repressed could be a technical challenge and therefore it is possible that not all nascent RNAs generated from Polycomb-repressed genes can be captured.

Single gene profiles

Single gene profiles are taken from UCSC genome browser (http://genome.ucsc.edu), using the following datasets:

- R-loop peaks, from Sanz et al. (2016), downloaded from GEO (GSM1720620);
- R-loops from the same paper, raw data downloaded from GEO and remapped as described below;
- GRO-seq for plus and minus strands from Jonkers et al. (2014), downloaded from GEO (GSE48895) as bedgraph files;
- H3K27me3, from Mikkelsen et al. (2007) (GSM307619), raw data downloaded from GEO and remapped as described below;
- H2AK119ub1, from Brookes et al. (2012) (GSM850471), raw data downloaded from GEO and remapped as described below;
- EZH2 and RING1B from Ku et al. (2008) (GSM327668 and GSM327669), raw data downloaded from GEO and remapped as described below;
- RNAPII-S5p, RNAPII-8WG16, RNAPII-S7p and RNAPII-S2p from Brookes et al., 2012 (GSM850467, GSM850469, GSM850468, GSM850470), raw data downloaded from GEO and remapped as described below;
- mRNA-seq from Brookes et al. (2012) (GSM850476), raw data downloaded from GEO and remapped as described below.

ChIP-seq sequenced reads were aligned to the mouse genome mm9 with Bowtie v2.0.5 (Langmead and Salzberg, 2012), with default parameters. Duplicated reads (i.e identical reads, aligned to the same genomic location) occurring more often than a threshold were removed. The threshold is computed for each dataset as the 95th percentile of the frequency distribution of reads. mRNA-seq reads were mapped to the mouse genome mm9 and the UCSC mm9 Known Gene GTF annotation file using TopHat (Kim et al., 2013) v2.0.8, default parameters.

p values and Statistical analysis

Statistical tests in all figures, except Figure 3D, were performed using two-tailed, unpaired Student's distribution t test, where *p < 0.05, **p < 0.01, ***p < 0.001. p value in Figure 3D is calculated with Wilcoxon rank-sum test. All numbers of independent biological repeats are indicated for each figure and panel in the corresponding Figure Legend.

DATA AND SOFTWARE AVAILABILITY

Datasets produced in this study have been deposited in GEO in the following link: https://www.ncbi.nlm.nih.gov/geo/query/acc.cgi?acc=GSE118115. Original images of western blot assays are available at Mendeley Data https://doi.org/10.17632/55f4vg9ww4.1.

Molecular Cell, Volume *73*

Supplemental Information

R-Loops Enhance Polycomb Repression

at a Subset of Developmental Regulator Genes

Konstantina Skourti-Stathaki, Elena Torlai Triglia, Marie Warburton, Philipp Voigt, Adrian Bird, and Ana Pombo

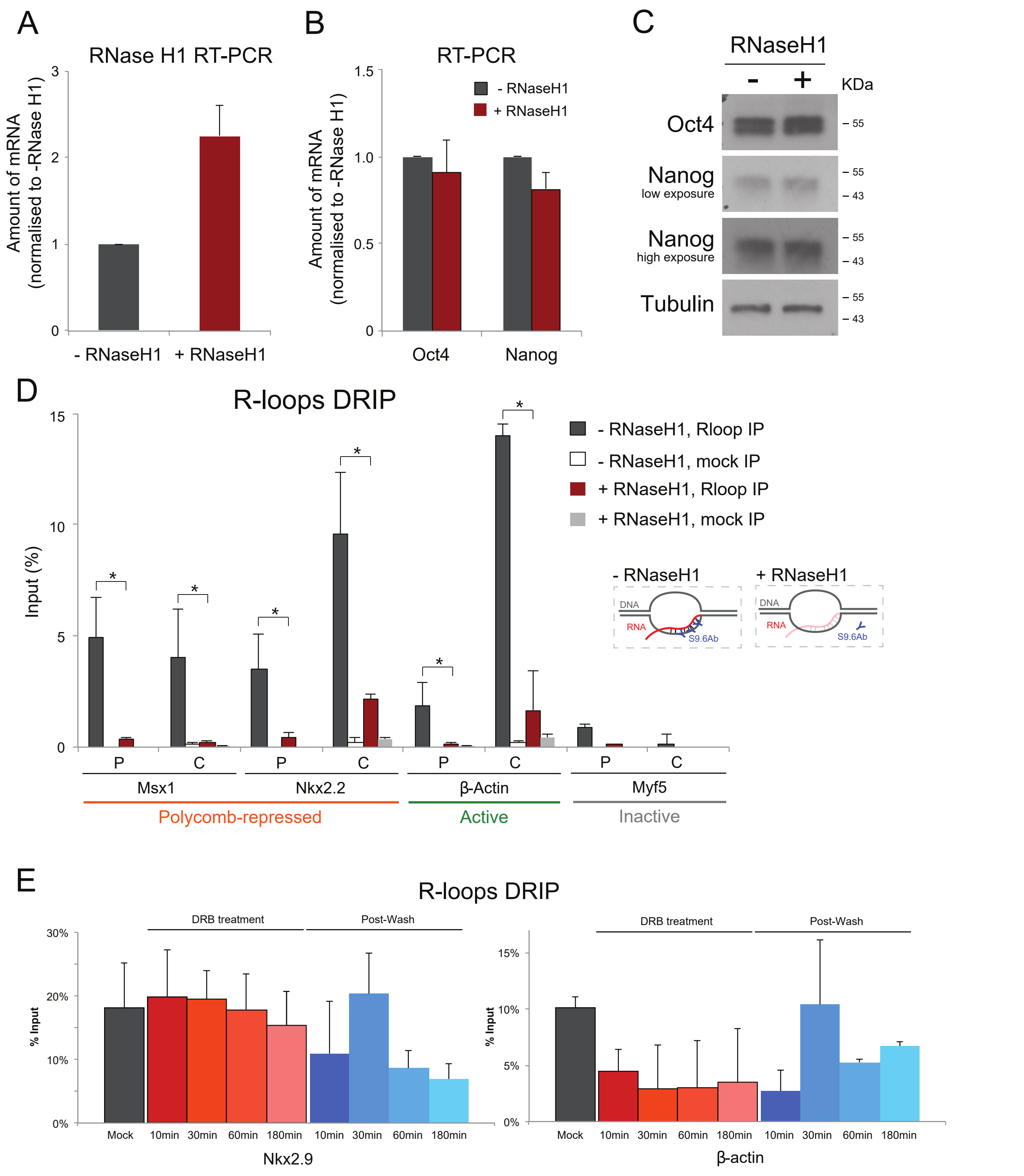

Figure S1. RNase H1 over-expression in mESCs resolves R-loops. (related to Figure 1)
(A-B) RT-qPCR analysis of RNase H1(A), Oct4 and Nanog (B) mRNA levels minus/plus RNase H1 over-expression. The amount of mRNA is normalised to control cells and was taken as 1. (C) Western blot analysis minus/plus RNase H1 over-expression. γ-tubulin was used as a loading control. (D) DRIP analysis following RNase H1 over-expression (red bars) on Polycomb-repressed, active and inactive genes. RT-qPCR and DRIP profiles are based on SD, n=3. Statistical significance was determined as in main figures. (E) DRIP analysis following DRB treatment and post-wash at the indicated time-points over the Polycomb-repressed gene *Nkx2.9* and the active *β-actin* gene. Error bars are SD, n=2.

Skourti-Stathaki_Fig.S2

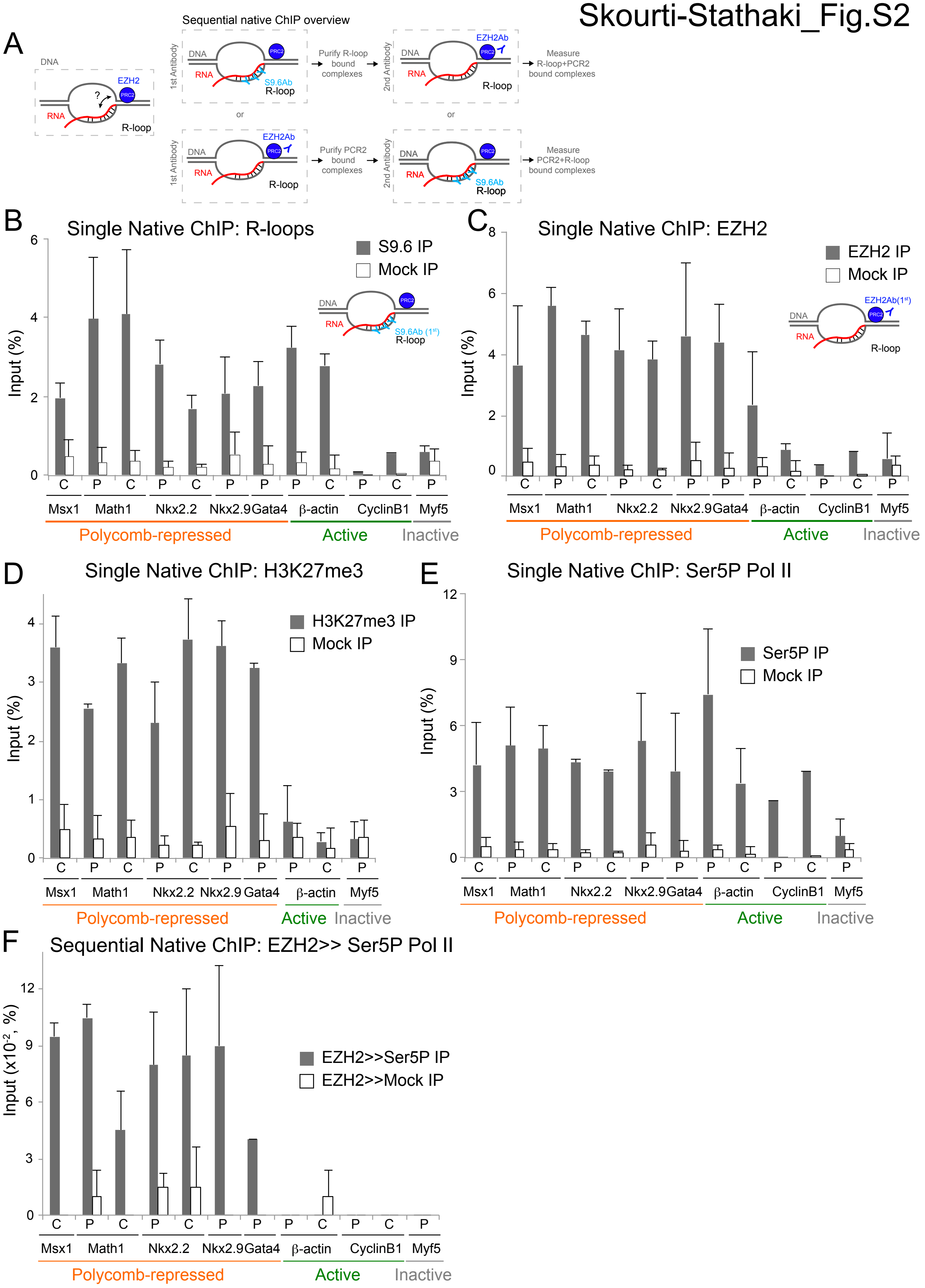

Figure S2. Validation of single native ChIP and sequential native ChIP analyses. (related to Figure 2)
(A) Schematic depicting the overview of the sequential native ChIP analysis. S9.6 (B) and EZH2 antibodies (C) native ChIP analyses on Polycomb-repressed, active and inactive genes. (D-E) Single native ChIP analyses on Polycomb-repressed genes, active and inactive genes, using H3K27me3 and Ser5P Pol II antibodies, respectively. **F.** Sequential native ChIP analysis of EZH2 with Ser5P Pol II. Error bars are SD, n=3.

Skourti-Stathaki_Fig.S3

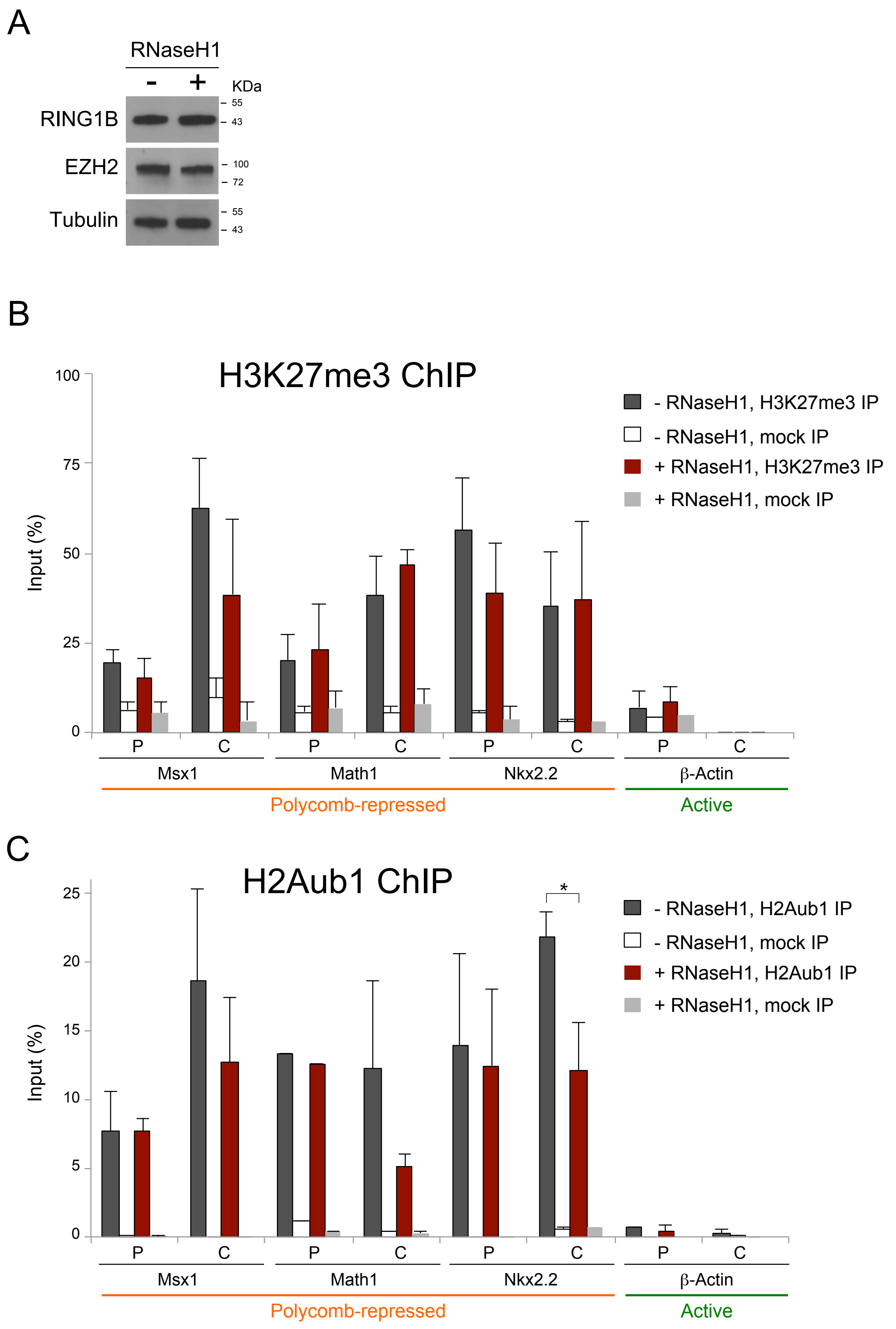

Figure S3. H3K27me3 and H2Aub1 levels upon RNase H1 over-expression. (related to Figure 2)
(A) Western blot analysis with/without RNase H1 over-expression. (B-C) ChIP analyses minus/plus RNase H1 over-expression on Polycomb-repressed and active genes, using H3K27me3 and H2Aub1 antibodies respectively. Error bars are SD, n=3 Statistical significance determined as in main figures.

Skourti-Stathaki_Fig.S4

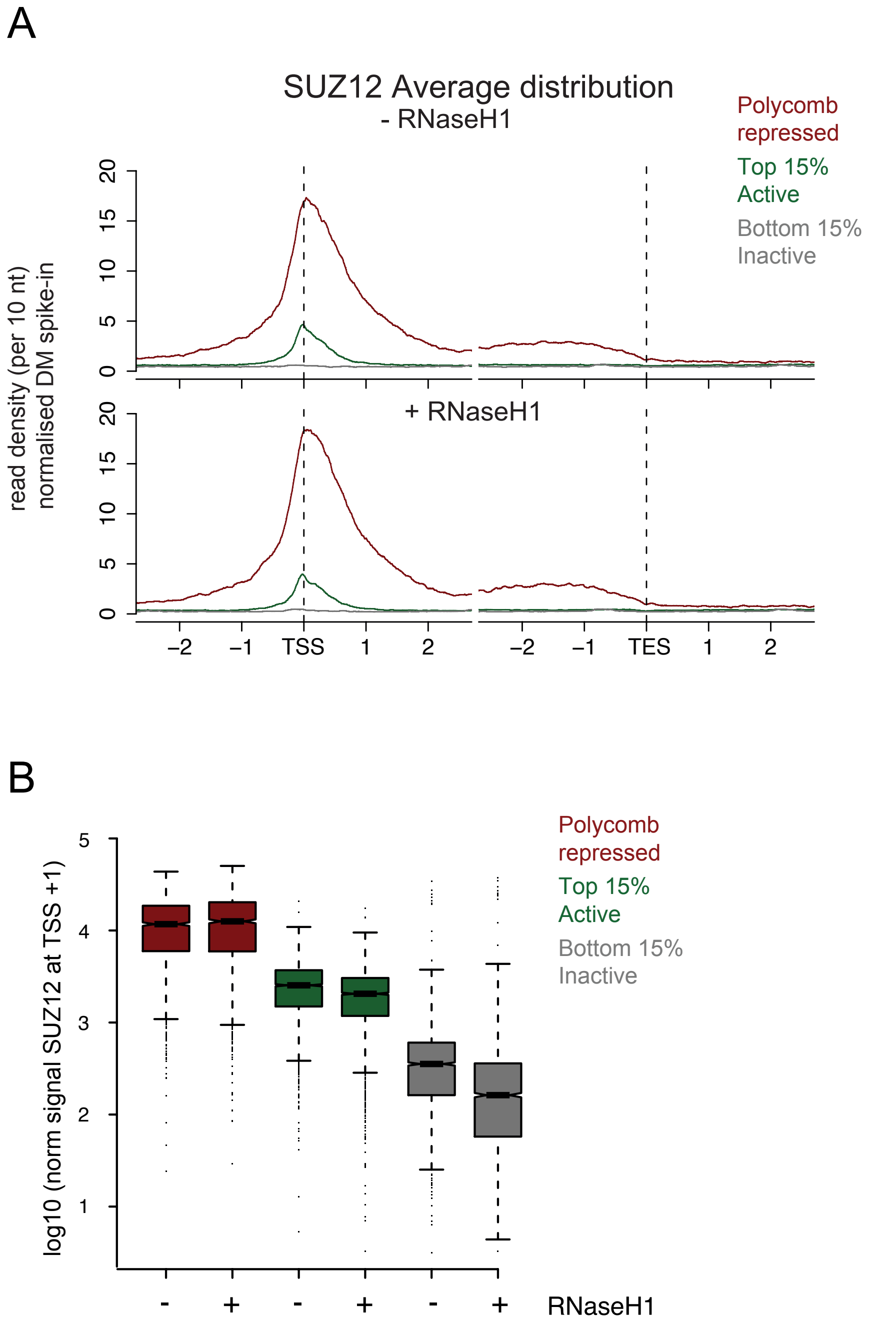

Figure S4. Genome-wide distribution of SUZ12 is only mildly affected upon R-loop resolution at Polycomb-repressed genes. (related to Figure 3)
(A) Average distribution of SUZ12 in minus/plus RNAseH1 at Polycomb-repressed genes (n=1632). Most active (Top 15%, n=2829) and least active genes (bottom 15% inactive, n=2829) are shown for comparison. (B) Boxplot with amount of signal for SUZ12 in absence (minus) or presence (plus) of RNAseH1 in 1kb centered around TSS at Polycomb repressed genes (n=1632). Most Active (Top 15%, n=2829) and least active genes (bottom 15% Inactive, n=2829) are shown for comparison. Amount of signal for both panels is normalized using Drosophila Spike-Ins (see Methods)

Skourti-Stathaki_Fig.S5

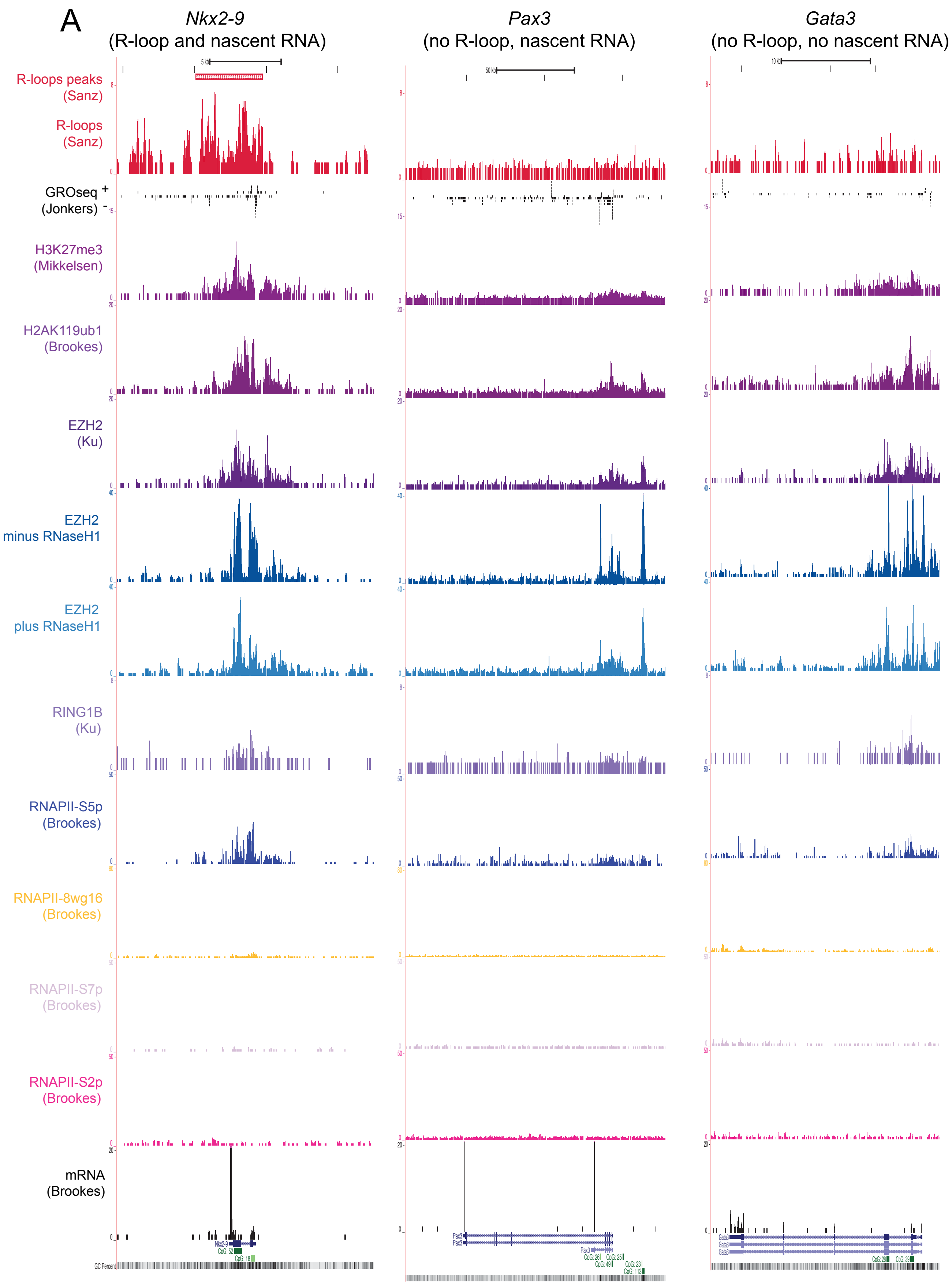

Figure S5. Representative examples of R-loop positive and R-loop negative Polycomb-repressed genes. (related to Figure 3)

UCSC browser tracks of R-loops, Polycomb, RNA Pol II and transcription at Polycomb-repressed genes with nascent RNA plus R-loops (*Nkx2.9*), nascent RNA minus R-loops (*Pax3*) and neither nascent RNA nor R-loops (*Gata3*).

Skourti-Stathaki_Fig.S6

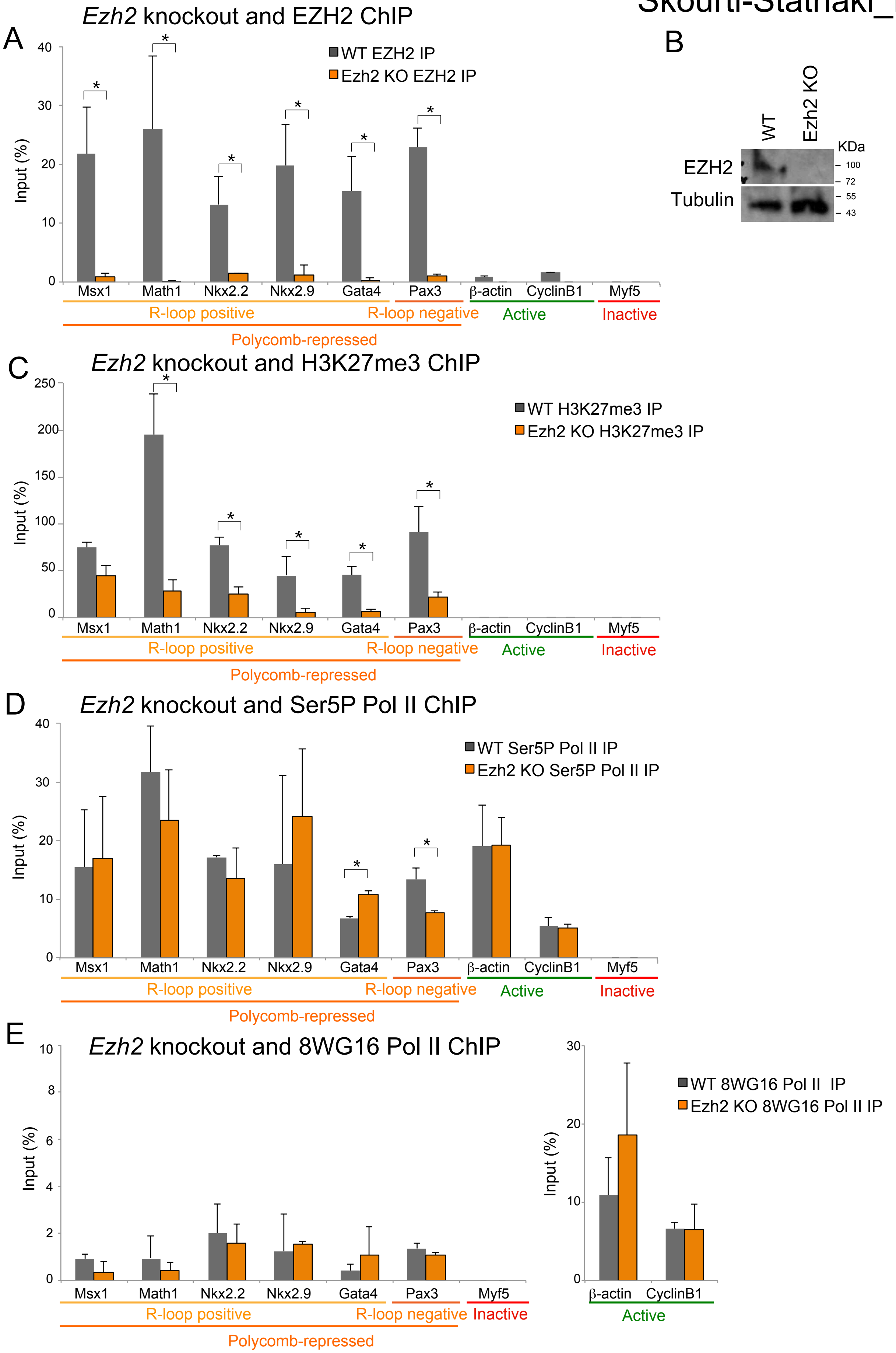

Figure S6. EZH2, H3K27me3, Ser5P and 8WG16 Pol II levels in *Ezh2* KO cells. (related to Figure 5)
(A) ChIP analysis in *Ezh2* KO cells (orange bars) using EZH2 antibody. (B) Western blot analysis in WT and *Ezh2* KO mESC probing for EZH2. γ-tubulin was used as a loading control. (C-E) ChIP analyses in *Ezh2* KO cells (orange bars) using H3K27me3 (C), Ser5P Pol II (D) and 8WG16 Pol II (E) antibodies. Error bars are SD, n=3. Statistical significance was determined as in main figures.

Skourti-Stathaki_Fig.S7

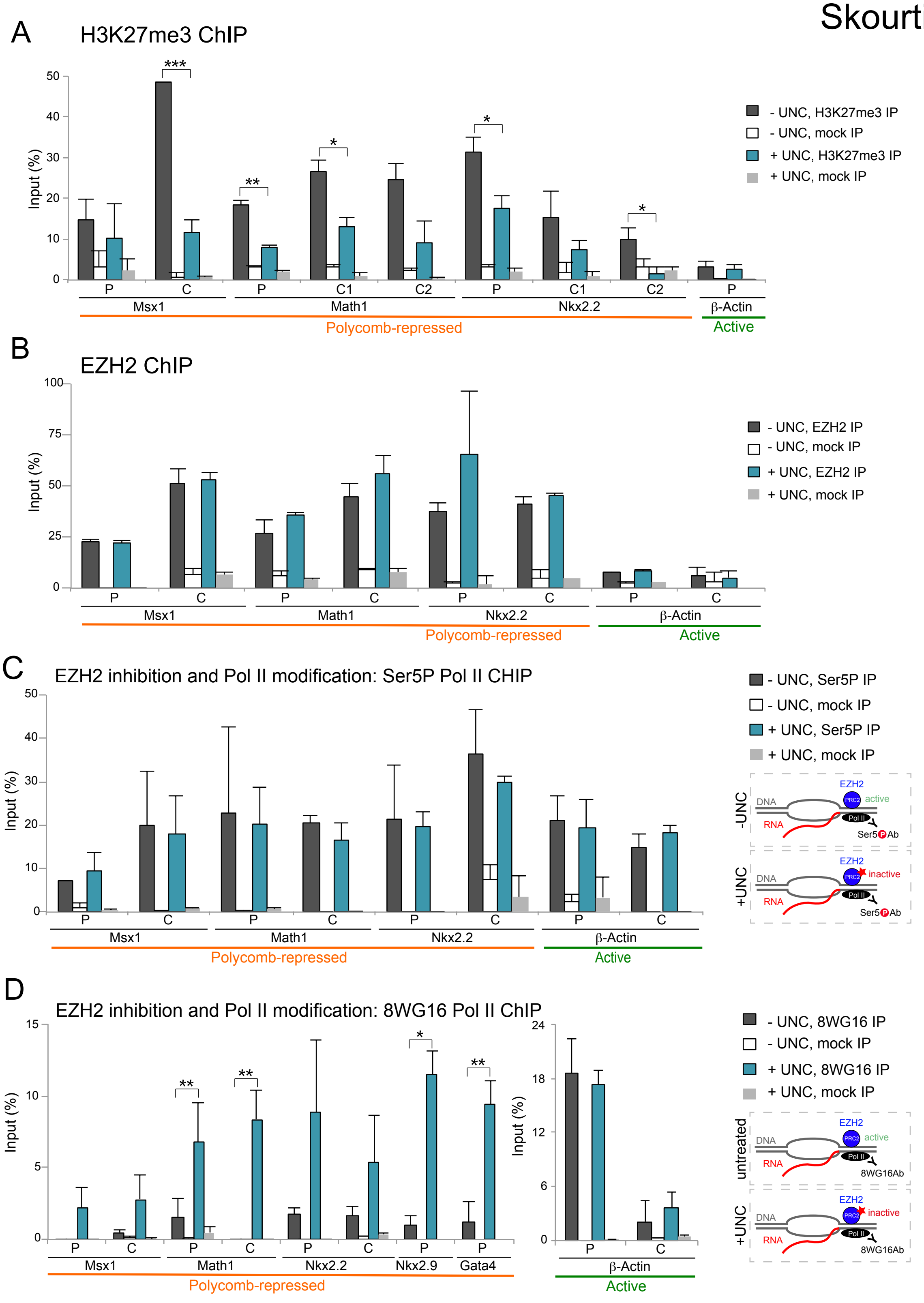

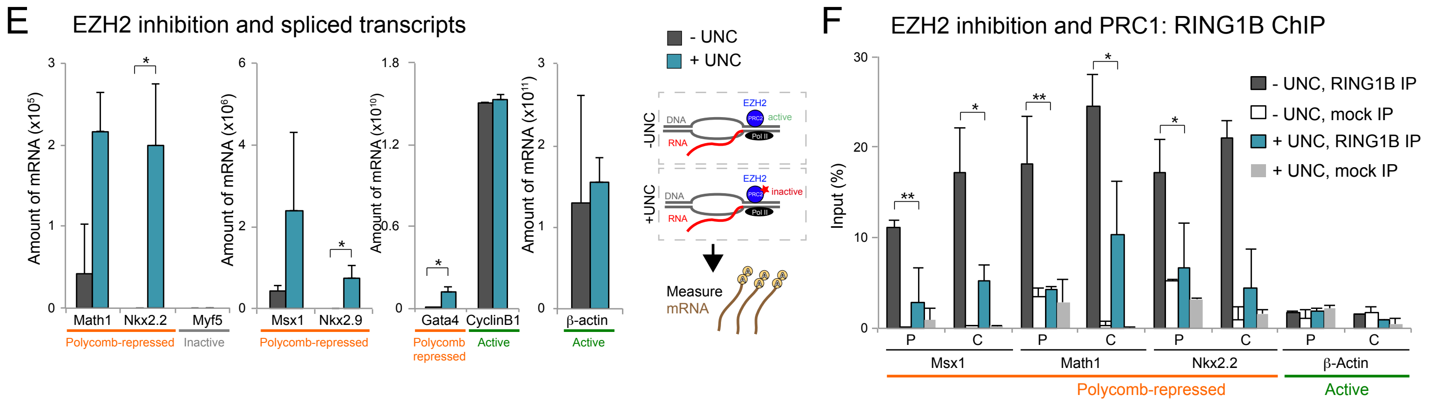

Figure S7. H3K27me3, EZH2, Ser5P, 8WG16 Pol II, mRNA and RING1B levels upon EZH2 catalytic inhibition. (related to Figure 6). (A-F). ChIP (A, B, C, D, F) and mRNA (E) analyses upon UNC treatment. Antibodies used for ChIP are indicated. Error bars are SD, n=3. Statistical significance was determined as in main figures.

Supplementary Table 1: Oligonucleotides (related to STAR Methods)

**Name**	**Sequence (5’ → 3’)**
**Polycomb-repressed genes**	
Msx1 P (F)	CTT AGC TAG GCG GAA AAG CTC
Msx1 P (R)	GAG AGA ACC ATT GGG CTG TG
Msx1 C1 (F)	ATC CTA GCT CTG CGG AGT TTC
Msx1 C1 (R)	TCC CCT CTT GCT AAA TCA TCC
Msx1 C2 (F)	AAA CCT GGG TGA CTT TGG ACT
Msx1 C2 (R)	AGC AGA GAC AGT GCC AAC CTA
Math1 P (F)	GGT CAG AGG AGG AAG GAA AAA
Math1 P (R)	CCC CCA ACT CTT TTA CCT CAG
Math1 C (F)	GTG AAT GGG GTA CAG AAG CAA
Math1 C (R)	TTG ATG TAG ATC TGG GCC ATC
Nkx2.2 P (F)	TAG ATA AAG GCG GGT GTT GAA
Nkx2.2 P (R)	CAG GAG ACT CAC CCC TCA AA
Nkx2.2 C1 (F)	TAC CAG CAA GGG GAG TTC TTT
Nkx2.2 C1 (R)	CCT CAT CCT CCA CAC CTA CAA
Nkx2.2 C2 (F)	TCT TCC TCA GCA TCT CCT CAA
Nkx2.2 C2 (R)	CGG TTT TGA AAT GCT GGT TTA
Nkx2.9 P (F)	TGG CAC CTT CCG GAC TTG
Nkx2.9 P (R)	AAG TGC GAG GCG CTC G
Gata4 P (F)	AAG AGC GCT TGC GTC TCT A
Gata4 P (R)	TTG CTA GCC TCA GAT CTA CGG
Msx1 nascent (F)	CGC TCG AGT TGG CCT TCT
Msx1 nascent (R)	CGG AGT CCT CCA CTT TGA CAC
Math1 nascent (F)	TGT GCG ATC TCC GAG TGA
Math1 nascent (R)	CTC GGA GGT GCC GTG TTA
Nkx2.2 nascent (F)	CGC TGC GCA GAC TCT CCT CT
Nkx2.2 nascent (R)	GAA GAG AAG CGC ATC AGG CG
Nkx2.9 nascent (F)	GTG CGC AGC CTC CTG AAT
Nkx2.9 nascent (R)	GGT CCC TCC TCC GCA CTC
Gata4 nascent (F)	GGA CTC ACG GAG ATC GCG
Gata4 nascent (R)	GGA CTC GGG GAA CCC TAC C
Msx1 spliced (F)	GCC TCT CGG CCA TTT CTC AG
Msx1 spliced (R)	CGG TTG GTC TTG TGC TTG CG
Math1 spliced (F)	GGA GAA GCT TCG TTG CAC GC
Math1 spliced (R)	GGG ACA TCG CAC TGC AAT GG
Nkx2.2 spliced (F)	TGT GCA GAG CCT GCC CCT TAA
Nkx2.2 spliced (R)	GCC CTG GGT CTC CTT GTC AT
Nkx2.9 spliced (F)	GGC CAC CTC TGG ACG CCT CG

(continues - part 2 of 3)	
Nkx2.9 spliced (R)	GCC AGC TGC GAC GAG TCT GC
Gata4 spliced (F)	GAG GCT CAG CCG CAG TTG CAG
Gata4 spliced (R)	CGG CTA AAG AAG CCT AGT CCT TGC TT
Pax3 P (F)	ACC TGT CCA CCC TTC TCT TGA
Pax3 P (R)	TCA CCC AAA GCT TGA TCA GGA
Pax3 nascent (F)	TCC CCA ACC CTT GCC TAC TAT
Pax3 nascent (R)	ATT GAG CGA TCG GAA TGA GGT
Pax3 spliced (F)	GTC CCA TGG TTG CGT CTC TAA
Pax3 spliced (R)	CTA AAC ATG CCC GGG TTC TCT
Hoxa7 P (F)	GAG AGG TGG GCA AAG AGT GG
Hoxa7 P (R)	CCG ACA ACC TCA TAC CTA TTC CTG
Hoxa7 nascent (F)	TAG ATC TTC GGG GAA CTT GGC
Hoxa7 nascent (R)	CAG AGT AGC CTT GGC CTT TCA
Hoxa7 spliced (F)	GGA AGC TGA GAG ACG TTG ACT
Hoxa7 spliced (R)	ATT TGT TGT CCG GCA GCT TTC
Mogat1 P (F)	TCC CTT TGC CTG TAG ACC TCT
Mogat1 P (R)	TCT GGC AAA AGC TCC CAA AAG
Mogat1 nascent (F)	GAC ACC ATG ACC ACA GCT CTT
Mogat1 nascent (R)	AGA TGC TCA AGT CAC ACC CAG
Mogat1 spliced (F)	GCA AGG AGG CAG AAG ATG GAA
Mogat1 spliced (R)	TCC AGG CAC GAA TAT TCC ATG A
**Active genes**	
β-actin P (F)	GAG GGG AGA GGG GGT AAA
β-actin P (R)	GAA GCT GTG CTC GCG G
β-actin C (F)	CAC CAT TCA CCA TCT TGT C
β-actin C (R)	TGA TCC ACA TCT GCT GG
CyclinB1 P (F)	GCT AGC TTG GAC AGC ACA CA
CyclinB1 P (R)	GTT CCC GTA GAA TGC GTT TC
CyclinB1 C (F)	AGT TTA GAG CCA GCC AGG ACT
CyclinB1 C (R)	GAG AAA AGC ACT GCA ATC AGG
β-actin nascent (F)	CCA CCC GCG AGC ACA
β-actin nascent (R)	CCG GCG TCC CTG CTT AC
β-actin spliced (F)	TCT TTG CAG CTC CTT CGT TG
β-actin spliced (R)	ACG ATG GAG GGG AAT ACA GC
CyclinB1 spliced (F)	TAG GGT GTC TTC TCG AAT CGG
CyclinB1 spliced (R)	ACC AAT GTC TCC AAG AGC AGT
RNase H1 (F)	GAA GGC ACA AGT GCA AGA CTC
RNase H1 (R)	TGT TCC TTC AAG GTG ATC CAG
Oct4 (F)	ACC TCA GGT TGG ACT GGG CCT A

(continues - part 3 of 3)	
Oct4 (R)	GCC TCG AAG CGA CAG ATG GT
Nanog (F)	GAA ATC CCT TCC CTC GCC ATC
Nanog (R)	CTC AGT AGC AGA CCC TTG TAA GC
**Inactive gene**	
Myf5 P (F)	GGT TGT GGT GGG ATA TGC TAA
Myf5 P (R)	GGA GTT TGG GAC TGT CTC TCT G
Myf5 C (F)	TCT GTG AGA TGG ATG GGA ACT
Myf5 C (R)	TCT TTG TGT CCC TCT CAG GTG
Myf5 nascent (F)	GGA ATA TAT AAA GAG CCC CAA CC
Myf5 nascent (R)	TTT GGG ACT GTC TCT CTG TAA TTA AC
Myf5 spliced (F)	GAT TGC TTG TCC AGC ATT GT
Myf5 spliced (R)	AGT GAT CAT CGG GAG AGA GTT
